# Pho4 Is Essential for Dissemination of *Cryptococcus neoformans* to the Host Brain by Promoting Phosphate Uptake and Growth at Alkaline pH

**DOI:** 10.1128/mSphere.00381-16

**Published:** 2017-01-25

**Authors:** Sophie Lev, Keren Kaufman-Francis, Desmarini Desmarini, Pierre G. Juillard, Cecilia Li, Sebastian A. Stifter, Carl G. Feng, Tania C. Sorrell, Georges E. R. Grau, Yong-Sun Bahn, Julianne T. Djordjevic

**Affiliations:** a Fungal Pathogenesis Laboratory, Centre for Infectious Diseases and Microbiology, The Westmead Institute for Medical Research, Westmead, NSW, Australia; b Marie Bashir Institute for Infectious Diseases and Biosecurity, The University of Sydney, Sydney, NSW, Australia; c Immunology and Host Defense Group, Department of Infectious Diseases and Immunology, Sydney Medical School, The University of Sydney, Sydney, NSW, Australia; d Mycobacterial Research Program, The Centenary Institute, Camperdown, NSW, Australia; e The Westmead Clinical School, Sydney Medical School, The University of Sydney, Westmead, NSW, Australia; f Vascular Immunology Unit, Department of Pathology, School of Medical Sciences, The University of Sydney, Sydney, NSW, Australia; g Department of Biotechnology, College of Life Science and Biotechnology, Yonsei University, Seoul, Republic of Korea; Carnegie Mellon University

**Keywords:** cryptococcal meningitis, cryptococcoma, *Cryptococcus neoformans*, fungal pathogenesis, HLH-type transcription factor, intravenous inoculation, murine models of cryptococcosis, phosphate-sensing and acquisition (PHO) pathway, signal transduction pathway

## Abstract

Phosphate acquisition by fungi is regulated by the phosphate-sensing and acquisition (PHO) signaling pathway. *Cryptococcus neoformans* disseminates from the lung to the brain and is the commonest cause of fungal meningitis worldwide. To investigate the contribution of PHO signaling to cryptococcal dissemination, we characterized a transcription factor knockout strain (*hlh3Δ/pho4Δ*) defective in phosphate acquisition. Despite little similarity with other fungal Pho4 proteins, Hlh3/Pho4 functioned like a typical phosphate-responsive transcription factor in phosphate-deprived cryptococci, accumulating in nuclei and triggering expression of genes involved in phosphate acquisition. The *pho4*Δ mutant strain was susceptible to a number of stresses, the effect of which, except for alkaline pH, was alleviated by phosphate supplementation. Even in the presence of phosphate, the PHO pathway was activated in wild-type cryptococci at or above physiological pH, and under these conditions, the *pho4*Δ mutant had a growth defect and compromised phosphate uptake. The *pho4Δ* mutant was hypovirulent in a mouse inhalation model, where dissemination to the brain was reduced dramatically, and markedly hypovirulent in an intravenous dissemination model. The *pho4Δ* mutant was not detected in blood, nor did it proliferate significantly when cultured with peripheral blood monocytes. In conclusion, dissemination of infection and the pathogenesis of meningitis are dependent on cryptococcal phosphate uptake and stress tolerance at alkaline pH, both of which are Pho4 dependent.

**IMPORTANCE** Cryptococcal meningitis is fatal without treatment and responsible for more than 500,000 deaths annually. To be a successful pathogen, *C. neoformans* must obtain an adequate supply of essential nutrients, including phosphate, from various host niches. Phosphate acquisition in fungi is regulated by the PHO signaling cascade, which is activated when intracellular phosphate decreases below a critical level. Induction of phosphate acquisition genes leads to the uptake of free phosphate via transporters. By blocking the PHO pathway using a Pho4 transcription factor mutant (*pho4Δ* mutant), we demonstrate the importance of the pathway for cryptococcal dissemination and the establishment of brain infection in murine models. Specifically, we show that reduced dissemination of the *pho4Δ* mutant to the brain is due to an alkaline pH tolerance defect, as alkaline pH mimics the conditions of phosphate deprivation. The end result is inhibited proliferation in host tissues, particularly in blood.

**Podcast**: A podcast concerning this article is available.

## Abstract (precis)

Cryptococcal meningitis is fatal without treatment and responsible for more than 500,000 deaths annually. To be a successful pathogen, *C. neoformans* must obtain an adequate supply of essential nutrients, including phosphate, from various host niches. Phosphate acquisition in fungi is regulated by the PHO signaling cascade, which is activated when intracellular phosphate decreases below a critical level. Induction of phosphate acquisition genes leads to the uptake of free phosphate via transporters. By blocking the PHO pathway using a Pho4 transcription factor mutant (*pho4Δ* mutant), we demonstrate the importance of the pathway for cryptococcal dissemination and the establishment of brain infection in murine models. Specifically, we show that reduced dissemination of the *pho4Δ* mutant to the brain is due to an alkaline pH tolerance defect, as alkaline pH mimics the conditions of phosphate deprivation. The end result is inhibited proliferation in host tissues, particularly in blood.

## INTRODUCTION

Phosphorus in the form of inorganic phosphate (P_i_) is essential for synthesis of membrane phospholipids and nucleic acids, energy storage and transfer (as a component of ATP), and signal transduction (via protein phosphorylation). Maintenance of intracellular P_i_ at high (millimolar) levels is required for normal cellular function (1). Fungi possess a phosphate-sensing and acquisition (PHO) pathway, which is absent in human cells. This pathway consists of a core signaling cascade: the cyclin-dependent kinase (CDK)-cyclin complex, the CDK inhibitor, and a transcriptional regulator(s). When intracellular phosphate is low, the signaling cascade induces expression of effector genes involved in P_i_ acquisition (reviewed in reference 2). These effector genes encode phosphate transporters and a variety of enzymes that mobilize P_i_ from complex organic sources (2).

The opportunistic fungal pathogen *Cryptococcus neoformans* causes life-threatening meningitis primarily in immunocompromised individuals and is a major cause of morbidity and mortality worldwide (3–5). *C. neoformans* initially infects the lungs, and in immunocompetent hosts, it grows as dense clusters known as cryptococcomas. The microenvironment within the cryptococcoma is more acidic than the surrounding tissues and blood (6), which are mildly alkaline. *C. neoformans* can also be transported via the circulation system to other organs, including the brain. Once in the tissues, it typically grows as cryptococcomas similar to those in the lung. Within each of its infection niches, *C. neoformans* must acquire sufficient nutrients, including phosphate, to sustain its growth and tolerate host-derived stress, including alkaline pH.

A core PHO signaling cascade comprised of the CDK Pho85, the cyclin Pho80, the CDK inhibitor Pho81, and the transcriptional regulator Pho4 has been identified in *C. neoformans* (serotype D) (7, 8). However, despite this signaling cascade being integral to the regulation of numerous effector genes, its role in cryptococcal fitness, stress tolerance, virulence, and organ-specific disease (lung and central nervous system) has not been studied. To date, only a few PHO pathway effector genes have been shown to play a role in the virulence of *C. neoformans*, as assessed in mouse inhalation models: the secreted and vacuolar acid phosphatase Aph1 (9) and the high-affinity phosphate transporters (Pho84, Pho89, and Pho840) (8). Although a mutant strain deficient in phosphate transport (*pho84*Δ *pho840*Δ *pho89*Δ mutant) was hypovirulent in a mouse inhalation model, the mutant colonized the lung and disseminated to the brain to the same extent as the wild type (WT) did (8).

Here we investigate the contribution of the PHO signaling cascade to the ability of *C. neoformans* to establish infection in murine inhalation and dissemination models. We focus predominantly on the effect of blocking the pathway using a *PHO4* deletion mutant (*pho4*Δ mutant) identified from a transcription factor knockout library screen. We also investigate the contribution of Pho4 to the ability of *C. neoformans* to grow and take up phosphate at physiological pH. We propose that by promoting phosphate uptake within the alkaline pH environment of the host, particularly in the blood, Pho4 plays a key role in growth, stress protection, and dissemination of *C. neoformans* to the central nervous system.

## RESULTS

### Identification of the PHO pathway transcriptional regulator (Pho4) in *C. neoformans*.

Due to the lack of transcription factor sequence conservation between *Saccharomyces cerevisiae* and *C. neoformans* (10), cryptococcal Pho4 was not identified in a BLAST search using fungal Pho4 sequences as a query. In the *C. neoformans* serotype D strain B4500 (JEC21), Pho4 was identified from a T-DNA insertion library screen after the present study had commenced (7). We used a similar approach to identify Pho4 in the serotype A strain H99, which involved screening a library of *C. neoformans* H99 transcription factor mutants constructed by Jung et al. (10). Given our recent finding that Aph1 (designated Pho5 in *S. cerevisiae* [Table 1]) is the sole source of phosphate-repressible extracellular acid phosphatase activity in *C. neoformans*, we reasoned that mutants lacking the *PHO4* gene would exhibit impaired Aph1 secretion during growth in phosphate-free medium (9). Cryptococcal strains were screened for Aph1 activity by assessing the hydrolysis of the chromogenic acid phosphatase substrate *p*-nitrophenyl phosphate (pNPP). This revealed that only one out of the total 155 transcription factor knockout strains failed to secrete Aph1. This knockout strain is referred to as *hlh3*Δ by Jung et al. (10), and the deleted gene corresponds to CNAG_06751 in the H99 genomic database.

**TABLE 1:** List of *C. neoformans* genes investigated in this study and their orthologs in *S. cerevisiae^a^*

*S. cerevisiae* gene	*S. cerevisiae* gene product description	*C. neoformans* locus tag (CNAG no.)	*C. neoformans* gene designation	*C. neoformans* gene product description (reference)
*PHO4*	Basic helix-loop-helix transcription factor; activates transcription cooperatively with Pho2p in response to phosphate limitation	CNAG_06751	*PHO4*/*HLH3*	Phosphate-responsive transcription factor (7)
*PHO84*	High-affinity inorganic phosphate (P_i_) transporter	CNAG_02777	*PHO84*	Phosphate/H^+^ symporter (8)
		CNAG_05459	*PHO840*	P_i_ transporter A-1 (8)
*PHO89*	Plasma membrane Na^+^/P_i_ cotransporter	CNAG_05075	*PHO89*	Sodium-dependent phosphate transporter (8)
*PHO5*	Phosphate-repressible acid phosphatase	CNAG_02944	*APH1*	Secreted and vacuolar acid phosphatase (9)
		CNAG_06967	*APH2*	Phytase
		CNAG_02681	*APH3*	Phytase
		CNAG_06115	*APH4*	Acid phosphatase
*VTC4*	Vacuolar transporter chaperone	CNAG_01263	*VTC4*	Vacuolar transporter chaperone 4 (8)
NA		CNAG_02353	*BTA1*	Betaine lipid (DGTS) synthase
*PHO81*	Cyclin-dependent kinase inhibitor	CNAG_02541	*PHO81*	Cyclin-dependent protein kinase inhibitor (7)
*GDE1*	Glycerophosphocholine phosphodiesterase	CNAG_06614	*GDE2*	Glycerophosphodiesterase

a NA, not applicable; DGTS, diacylglycerol-trimethylhomoserine.

The predicted *C. neoformans* Hlh3 (*Cn*Hlh3) protein is 795 amino acids long and contains a basic helix-loop-helix (HLH) domain similar to that of *S. cerevisiae* Pho4 (*Sc*Pho4) (see Fig. S1A in the supplemental material). However, the overall similarity between *Sc*Pho4 and *Cn*Hlh3 is only 27%, explaining why no hits were obtained in the BLAST search. Hlh3 showed a higher degree of sequence similarity to the *Candida albicans* Pho4 homolog (50%) and, as established during the conduct of our study, 90% identity to Pho4 from *C. neoformans* serotype D (7). On the basis of the protein nomenclature in *S. cerevisiae*, we will refer to Hlh3 as Pho4 hereafter. We also performed phylogenetic analysis of the fungal Pho4 homologs using only their HLH domains for alignment, as overall similarity among Pho4 proteins is low (Fig. S1B). Figure S1C demonstrates that cryptococcal Pho4 proteins are evolutionarily distant from their counterparts in other fungi. However, all Pho4 proteins clustered together when compared with functionally different proteins containing the HLH domain. Using the *pho4**Δ*** mutant strain identified in the library screen, we constructed a *PHO4* reconstituted strain (*pho4Δ+PHO4* strain) as described in Materials and Methods (Fig. S2). We verified the correct genotype of the *pho4Δ* and *pho4Δ*+*PHO4* strains by performing Southern blotting (Fig. S3A to C).

10.1128/mSphere.00381-16.3 FIG S1  *C. neoformans* Pho4 (CnPho4) shares limited homology with its orthologs in other fungi. (A) Diagram representing domain organization and functional motifs in CnPho4. NES, nuclear export signal; NLS, nuclear localization signal; HLH, basic helix-loop-helix DNA binding domain. (B) Alignment of CnPho4 with other fungal Pho4 proteins. Due to low overall similarity, only the sequence of the HLH domain was used for the alignment. Species used for the alignment and NCBI accession numbers are as follows: *Pyrenophora tritici-repentis* EDU50072 (Ptr_EDU50072), *Aspergillus fumigatus* XP_747924 (Af_XP_747924), *Saccharomyces cerevisiae* KZV11639 (Sc_Pho4), *Candida albicans* KHC37397 (Ca_KHC37397), *Neurospora crassa* AAA33603 (Nc_AAA33603), *Fusarium oxysporum* EMT61942 (Fo_EMT61942), *Cryptococcus neoformans* var. *grubii* H99 XP_012047121 (CnA_CNAG_06751), and *Cryptococcus neoformans* var. *neoformans* JEC21 XP_569062 (CnD_CNB00520). (C) Phylogenetic tree of fungal Pho4 proteins based on the alignment of their HLH DNA binding domains. Bootstrap values are indicated. *S. cerevisiae* Tye7 (Sc_Tye7) and Cbf1 (Sc_Cbf1) represent transcription factors that contain an HLH domain but that are not involved in phosphate homeostasis. Accession numbers: Sc_Tye7, NP_014989; Sc_Cbf1, NP_012594. Download FIG S1, PDF file, 0.2 MB. Copyright © 2017 Lev et al. 2017 Lev et al. This content is distributed under the terms of the Creative Commons Attribution 4.0 International license.

10.1128/mSphere.00381-16.4 FIG S2  *PHO4* reconstitution in the *pho4*Δ mutant restores secretion of acid phosphatase (Aph1) (A) and expression of *PHO4* (B). (A) To screen for successful integration of the *PHO4-NEO* construct into the *pho4*Δ mutant, Geneticin-resistant transformants were inoculated into phosphate-deficient medium (MM-KCl) in a 96-well plate. pNPP substrate was then added for 5 min, and the reaction was stopped by adding 50 μl of saturated Na_2_CO_3_. WT H99 was included as a positive control and is indicated by the arrow (the yellow color indicates the presence of acid phosphatase activity). The *pho4*Δ mutant was included as a negative control and is indicated by the star (the lack of color indicates no acid phosphatase activity). The rest of the wells contain potential *pho4*Δ+*PHO4* strains. (B) RNA was prepared from the strains indicated following their growth in phosphate-deficient (Pi-) (MM-KCl) and phosphate-replete (Pi+) (MM-KH_2_PO_4_) media. *PHO4* mRNA levels were then compared by qPCR after normalizing to the housekeeping gene *ACT1* as described in Materials and Methods. Download FIG S2, PDF file, 0.1 MB. Copyright © 2017 Lev et al. 2017 Lev et al. This content is distributed under the terms of the Creative Commons Attribution 4.0 International license.

10.1128/mSphere.00381-16.5 FIG S3  Verification of the *pho4*Δ+*PHO4* reconstituted strain. (A and B) Southern blot hybridization to confirm single integration of the *PHO4* deletion and reconstitution constructs using fragments of the nourseothricin (NAT) (A) and neomycin (NEO) (B) resistance cassettes as probes. Genomic DNA was prepared from each strain indicated and digested with HindIII. In panel A, using the NAT resistance probe, a single integration event was observed for the *PHO4-NAT* deletion construct in both *pho4Δ* and *pho4*Δ+*PHO4* strains. The smaller size of the NAT-hybridizing fragment observed in the reconstituted strain (5.4 kb) is due to single crossover integration of the full-length *PHO4-NEO* construct into the upstream region of the *PHO4* gene within the *Δpho4* mutant. This single integration event resulted in a smaller NAT-hybridizing fragment following genomic digestion with HindIII. In panel B, a single *PHO4-NEO* integration event is demonstrated in the reconstituted strain. (C) Diagram representing the genomic *PHO4* locus in WT, deletion mutant, and reconstituted strains. Integration of the *PHO4* reconstitution construct (*PHO4*-NEO^R^) upstream of the native *PHO4* was inferred based on the altered size of the NAT^R^-containing fragment. (D) Restoration of expression of the Pho4-dependent genes *APH1*, *PHO84*, and *PHO89* in the *pho4Δ+PHO4* reconstituted strain as assessed using qPCR. The indicated stains were grown overnight in YPD, washed twice with water, and resuspended in either phosphate-replete medium MM-KH_2_PO_4_ (Pi+) or phosphate-deficient medium MM-KCl (Pi-) and incubated at 30°C for 3 h. Cells were collected, and RNA was extracted for cDNA synthesis and qPCR. Gene expression was normalized to the housekeeping gene, actin (*ACT1*). Download FIG S3, PDF file, 0.2 MB. Copyright © 2017 Lev et al. 2017 Lev et al. This content is distributed under the terms of the Creative Commons Attribution 4.0 International license.

### Pho4 is phosphate responsive. (i) Pho4 is required for growth in the absence of phosphate.

**FIG 1:**
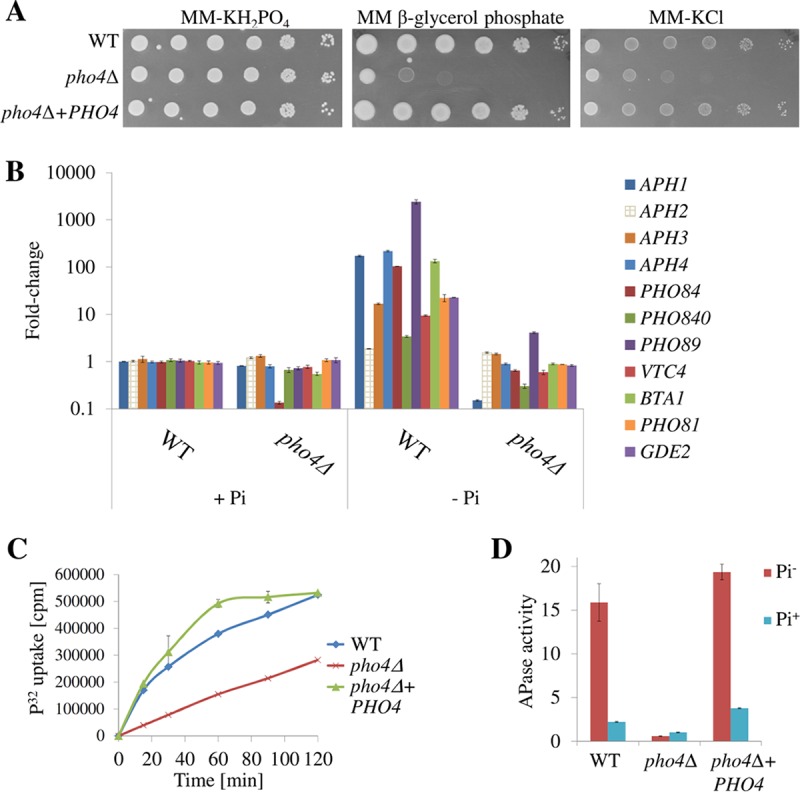
Pho4 promotes cryptococcal growth in the absence of free phosphate by inducing expression of genes involved in phosphate acquisition to allow optimal phosphate uptake. (A) Spot dilution assay with the indicated strains on minimal medium (MM) supplemented with 29.4 mM KH_2_PO_4_ (MM-KH_2_PO_4_), 29.4 mM β-glycerol phosphate, or 29.4 mM KCl (MM-KCl). The growth of the *pho4*Δ mutant was compromised when β-glycerol phosphate was the sole phosphate source and in the absence of free phosphate (MM-KCl). *PHO4* reconstitution recovers this phenotype. (B) Expression of genes involved in phosphate acquisition was tested in the WT and *pho4*Δ strains after 3 h of incubation in MM-KH_2_PO_4_ (with orthophosphate [+Pi]) or MM-KCl (−Pi). Expression data were normalized to the expression of the housekeeping gene, *ACT1* (CNAG_00483), and plotted as fold change relative to the value for the WT (+Pi). Results represent the mean fold changes plus standard deviations (SDs) (error bars). Results are a representative example of two biological replicates, each performed in technical triplicate. (C) Uptake of radioactive orthophosphate (P^32^) by phosphate-starved strains in MM. (D) Extracellular acid phosphatase (APase) activity after 4 h of incubation in MM with 5 mM KH_2_PO_4_ (Pi^+^) or in MM (Pi^−^). In panels C and D, the results represent the means ± SDs (*n* = 3 biological replicates).

Given that the Pho4 orthologs from *C. neoformans* and *S. cerevisiae* share low sequence similarity, we investigated whether cryptococcal Pho4 is required for growth in the absence of phosphate, as this was not tested in serotype D (7). In phosphate-replete minimal medium (MM-KH_2_PO_4_ [see Materials and Methods]), the WT, *pho4*Δ+*PHO4*, and *pho4*Δ strains all grew at similar rates (Fig. 1A). We then replaced free phosphate in the medium with β-glycerol phosphate. This complex source of phosphate is a preferred substrate of secreted acid phosphatase Aph1 (9). Figure 1A demonstrates that the *pho4*Δ mutant grew more slowly than the WT and *pho4*Δ+*PHO4* strains on β-glycerol phosphate, indicating that Pho4 is essential for the mobilization of phosphate from this source. Growth of the *pho4*Δ mutant in the absence of any exogenous source of phosphate (MM-KCl medium [see Materials and Methods]) was also assessed. Under these conditions, cells must mobilize phosphate from intracellular sources. Figure 1A demonstrates that the *pho4*Δ strain grew more slowly than the WT and *pho4*Δ+*PHO4* strains on MM-KCl, consistent with the reduced ability of this mutant to mobilize intracellular phosphate to meet its growth requirements.

### (ii) Pho4 induces expression of genes involved in phosphate acquisition.

To confirm that Pho4 is a phosphate-responsive transcription factor, we compared the expression of known and candidate Pho4 target genes involved in mobilization (e.g., phosphatases) and uptake (transporters) of phosphate in the WT and *pho4*Δ strains during phosphate deprivation. Expression of the high-affinity phosphate transporters, *PHO84*, *PHO840*, and *PHO89*, was markedly upregulated in the WT strain (104-fold, 3.4-fold, and 2,416-fold, respectively) in phosphate-deficient medium, as previously reported by Kretschmer et al. (8) and Toh-e et al. (7) in serotypes A and D, respectively. However, in the *pho4*Δ mutant, expression of *PHO84* and *PHO840* remained basal, and that of *PHO89* was only marginally increased (4-fold versus 2,416-fold in the WT) (Fig. 1B). Induction of *PHO84* and *PHO89* under phosphate-deficient conditions was restored in the *pho4*Δ+*PHO4* strain (Fig. S3D). In agreement with the lack of induction of the phosphate transporter genes, uptake of radioactive orthophosphate (^32^P_i_) by the phosphate-starved *pho4*Δ strain was impaired (Fig. 1C). However, the WT and *pho4*Δ strains took up ^32^P_i_ at a similar rate in phosphate-replete medium (yeast extract-peptone-dextrose [YPD] [see Materials and Methods]; ~2.5 mM free phosphate) (Fig. S4A), suggesting that the basal rates of P_i_ uptake are similar in the two strains under this condition and sufficient to support growth.

10.1128/mSphere.00381-16.6 FIG S4  Phosphate uptake and accumulation of polyphosphate chains (polyPs) are unaffected in the *pho4*Δ mutant grown in phosphate-replete medium (YPD). (A) Fungal cells from a YPD starter culture were resuspended in fresh YPD and supplemented with ^32^P_i_ (orthophosphate). After 5 h of incubation, the cells were pelleted and the amount of cell-associated ^32^P_i_ orthophosphate was determined by scintillation counting. (B) PolyPs were extracted from cells grown for 8 h in YPD broth. RNA (14 µg) was loaded onto a 3% metaphore gel, and RNA and polyPs were visualized by toluidine blue staining. 100-bp DNA ladder (M) and sodium phosphate glass type (P_45_) were used as markers to estimate the polyP size range. Download FIG S4, PDF file, 0.1 MB. Copyright © 2017 Lev et al. 2017 Lev et al. This content is distributed under the terms of the Creative Commons Attribution 4.0 International license.

In *C. neoformans*, Aph1 is the only acid phosphatase containing a leader peptide, which targets it to vacuoles and the cell exterior (9). Figure 1B demonstrates that when phosphate is absent, expression of *APH1* is highly induced in the WT strain (83-fold) and *pho4*Δ+*PHO4* strain (115-fold; Fig. S3D), but not in the *pho4Δ* mutant. As expected, extracellular acid phosphatase activity of the *pho4Δ* mutant was not increased under inducing conditions (Fig. 1D). In the absence of phosphate, two other predicted acid phosphatases were induced in the WT strain, but not in the *pho4*Δ mutant: CNAG_02681 (*APH3*) and CNAG_06115 (*APH4*) (17-fold and 218-fold, respectively). An additional acid phosphatase encoded by CNAG_06967 (*APH2*) was only marginally induced in the WT (1.9-fold) (Fig. 1B). Aph1, Aph2, Aph3, and Aph4 all contain the characteristic histidine phosphatase signature domain of the branch 2 histidine phosphatase superfamily. However, unlike Aph1, Aph2, Aph3, and Aph4 are predicted to be intracellular.

Expression of another gene, *VTC4*, was upregulated 9-fold in the WT strain in response to phosphate starvation (Fig. 1B) (8). Vtc4 is a polyphosphate polymerase and a subunit of the vacuolar transport chaperone complex. It is involved in the synthesis of polyphosphate chains (polyPs) at the vacuolar membrane. PolyPs are phosphate and energy storage molecules, but they also sequester toxic cations and influence blood coagulation (8, 11). The lack of Vtc4 impairs the ability of the fungal cells to promote blood coagulation, but not virulence (8). PolyPs are the first phosphate store to be utilized in WT yeast upon phosphate starvation and are rapidly hydrolyzed to supply free phosphate (12, 13; our unpublished observations). As the *vtc4*Δ mutant does not produce polyphosphates under phosphate-replete conditions (YPD medium) (8), we tested whether the *pho4*Δ mutant has the same defect. However, polyphosphate production in the *pho4*Δ mutant was not affected (Fig. S4B), suggesting that basal *VTC4* expression in the *pho4*Δ mutant is sufficient to allow polyphosphate synthesis under phosphate-replete conditions. Other Pho4-dependent genes include *PHO81*, *GDE2*, and* BTA1*, which encode a cyclin-dependent kinase inhibitor, a glycerophosphodiesterase involved in phospholipid remodeling, and a putative betaine lipid synthase, respectively (Fig. 1B). In contrast to its ortholog in serotype D (7), *PHO4* in serotype A was not upregulated during phosphate starvation, indicative of a lack of positive autoregulation (Fig. S2B).

### (iii) A cyclin-dependent kinase regulates Pho4. 

**FIG 2:**
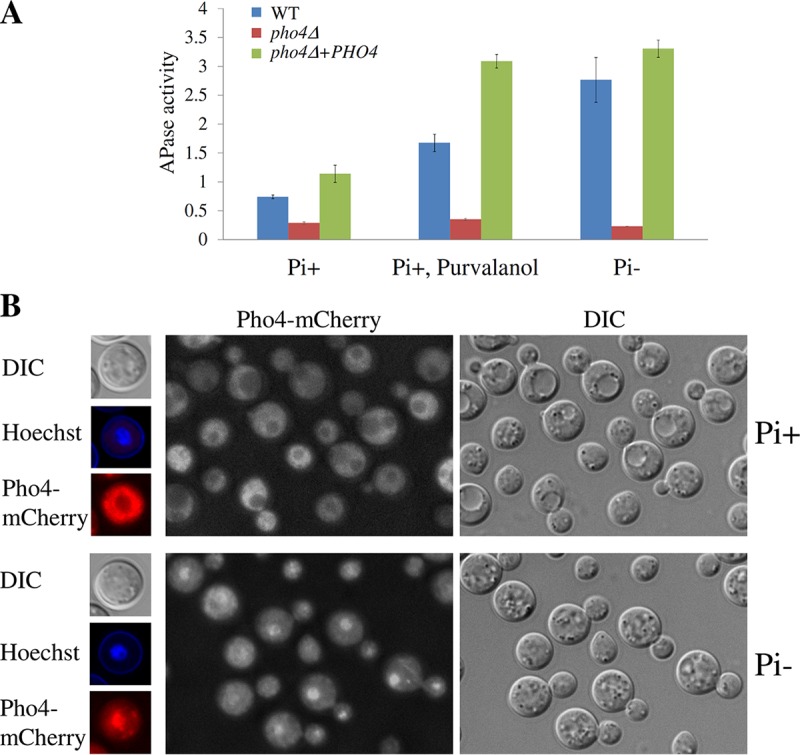
Pho4 is regulated by the cyclin-dependent kinase Pho85 and responds to phosphate deprivation by translocating to nuclei. (A) YPD-grown cells were incubated for 3 h in MM plus 1 mM KH_2_PO_4_ (MM Pi+) and dimethyl sulfoxide (DMSO) (negative control) or MM Pi+ with purvalanol A (50 μM). MM without KH_2_PO_4_ supplemented with DMSO served as a positive control (Pi−). Cell-associated APase activity, which served as a readout for Pho4 activation, was measured using the chromogenic substrate pNPP. Results represent the means ± SDs (*n* = 3 biological replicates). (B) Cells expressing Pho4-mCherry were incubated for 2 h in MM-KCl (Pi−) or MM-KH_2_PO_4_ (Pi+) and viewed using a DeltaVision fluorescence microscope. Nuclei were visualized with Hoechst stain (5 min, 25 µg/ml stain in the growth medium). DIC, differential interference contrast.

When P_i_ is abundant in *S. cerevisiae*, Pho4 is phosphorylated by the CDK Pho85 and exported from the nucleus to the cytoplasm. When P_i_ is deficient, Pho81 inactivates Pho85, allowing unphosphorylated Pho4 to remain in the nucleus to trigger the expression of effector genes (reviewed in references 2 and 14). To gain mechanistic insight into Pho4 activation in *C. neoformans*, we tested the effect of CDK inhibition on Pho4 activation under conditions where Pho4 is normally inactive (P_i_-replete medium). CDK (Pho85) inhibition was achieved using purvalanol A, and secreted acid phosphatase (APase) activity was used as the readout for Pho4 activation. As expected, APase activity was repressed in all strains grown in the presence of P_i _and induced in the WT and *pho4*Δ+*PHO4* strains, but not the *pho4*Δ mutant strain, in the absence of P_i_ (Fig. 2A). Following addition of purvalanol A to strains grown in the presence of P_i_, APase activity was induced in the WT and *pho4*Δ+*PHO4* strains, but not in the *pho4*Δ mutant strain. These results confirm that Pho4 is a target of CDK regulation, with Pho85-dependent phosphorylation of Pho4 preventing it from triggering gene expression (Fig. 2A).

### (iv) Phosphate deprivation triggers Pho4 translocation to nuclei. 

Pho4 from *C. neoformans* contains features of a transcription factor that shuttles in and out of the nucleus (Fig. S1A), namely, two nuclear localization signals (NLS) located on either side of the HLH domain at the C terminus and one nuclear export signal (NES) at the N terminus. To determine whether Pho4 responds to phosphate deprivation by translocating to nuclei, we replaced native *PHO4* with mCherry-labeled *PHO4* (*PHO4*-mCherry) under the control of the constitutive promoter (Fig. S5A to F). We confirmed that mCherry-tagged Pho4 (Pho4-mCherry) is functional by demonstrating that the WT and Pho4-mCherry strains grew at similar rates on medium containing β-glycerol phosphate as the sole source of phosphate (Fig. S5G). Anti-mCherry Western blot analysis of the Pho4-mCherry-expressing and WT strains grown under P_i_-depleted and P_i_-replete conditions confirmed that full-length Pho4-mCherry fusion protein is produced under both conditions (Fig. S5H). The absence of breakdown products confirmed that the fusion protein is stable (Fig. S5H). Figure 2B shows that Pho4-mCherry is cytosolic and excluded from the nuclei when the reporter strain is grown in the presence of phosphate. In the absence of phosphate, Pho4-mCherry localization is predominantly nuclear.

10.1128/mSphere.00381-16.7 FIG S5  Construction of the *C. neoformans* strain constitutively expressing Pho4-mCherry and its verification. (A) Diagram of the PHO4-mCherry-NEO construct. The 3′ end of the *PHO4* coding sequence minus the stop codon (encoding positions 1384 to 2495) and 1,153 bp of the genomic DNA downstream of *PHO4* (3′ flank) were amplified by PCR using the primers indicated by the red arrows and cloned into pCherry-NEO, creating PHO4-mCherry-NEO. The restriction sites used for cloning the two *PHO4* fragments are indicated. PHO4-mCherry-NEO was linearized with KpnI and introduced into the genome of the WT H99 strain using biolistic transformation. (B) Diagram of the genomic *PHO4* locus following integration of the PHO4-mCherry-NEO construct**.** Purple arrows indicate primers used to verify integration after biolistic transformation. Abbreviations: T_Hog1, Hog1 terminator used for expression of mCherry; ActP, actin promoter of NEO(R); NEO(R), aminoglycoside phosphotransferase gene encoding neomycin resistance; T_Trp, tryptophan terminator of NEO(R). (C) Introduction of a Gpd1 promoter in front of Pho4-mCherry. Gpd1p-PHO4-HYG construct for integration via double crossover was created using overlap PCR as described in Materials and Methods and used to transform PHO4-mCherry-NEO transformants created in panel B using biolistic transformation. From left to right, the genomic region upstream of *PHO4* (5′ flank), actin promoter of HYG(R) (ActP), the hygromycin B phosphotransferase gene conferring hygromycin B resistance [HYG(R)], Gal7 terminator of HYG(R) (T_Gal7), glycerol-3-phosphate dehydrogenase promoter (Gpd1p),* PHO4* coding sequence (*PHO4*), Hog1 terminator used for expression of mCherry (T_Hog1), actin promoter of NEO(R) (ActP), aminoglycoside phosphotransferase gene encoding neomycin resistance [NEO(R)], and tryptophan terminator of NEO(R) (T_Trp) are shown. The primers used to create and verify the construct are indicated by red and purple arrows, respectively, and are listed in Table S1. (D to H) Screening/verification of *PHO4*-mCherry-expressing *C. neoformans***.** (D) PHO4-mCherry-Neo construct integration. Genomic DNA prepared from six Geneticin-resistant clones was PCR amplified using the primer pair HLH3-int-s/ActP-a. The position of primer binding is indicated in panel B, and the primer sequences are listed in Table S1. The presence of a single band with the expected size of 2,255 bp in clones 2, 3, and 4, but not in the WT control, was indicative of successful recombination. (E and F) GPD1p-PHO4-HYG construct integration. Amplification across the 5′ (E) and 3′ (F) junctions with primer pairs Hlh3-5′-s/ActP-a and Gpd1p-int-s/Hlh3-int-a produced bands of the expected sizes (1,403 bp and 16,70 bp, respectively) in clones 7 and 8. Primer sequences are listed in Table S1. (G) Pho4-mCherry-expressing strain grows on β-glycerol phosphate as the sole source of phosphate, confirming that recombinant Pho4 is functional. (H) Western blot analysis of Pho4-mCherry**.** Pho4-mCherry-expressing and WT strains were incubated for 2 h in MM-KCl (Pi-) or MM-KH_2_PO_4_ (Pi+) medium, and total protein was extracted using TRIzol. Following SDS-PAGE, Pho4-mCherry was detected by Western blotting using anti-mCherry antibodies. Two Pho4-mCherry variants/isotypes were detected with molecular masses of 113 and 140 kDa (see arrows), indicative of the production of full-length fusion protein. The fusion protein is also stable, as indicated by the absence of breakdown products. Download FIG S5, PDF file, 0.4 MB. Copyright © 2017 Lev et al. 2017 Lev et al. This content is distributed under the terms of the Creative Commons Attribution 4.0 International license.

### Roles of Pho4 in virulence and disseminated infection. (i) Dissemination of the *pho4*Δ mutant to the brain is severely compromised in a mouse pulmonary infection model.

**FIG 3:**
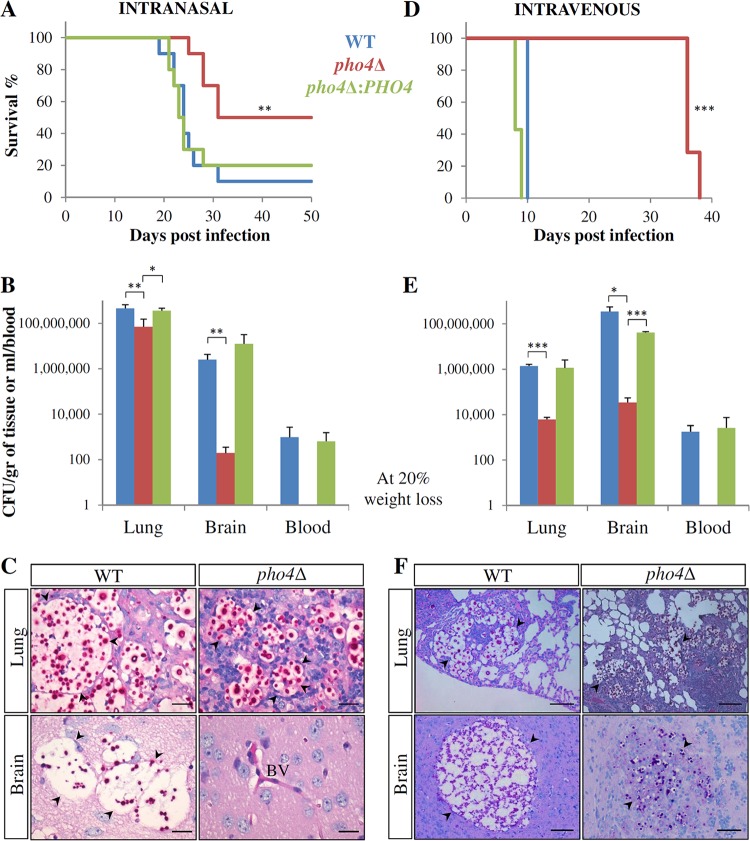
The *pho4*Δ mutant is less virulent than the WT strain in intranasal (A to C) and intravenous (D to F) models of cryptococcosis. (A) Survival of C57BL/6 mice infected intranasally with 5 × 10^4^ WT, *pho4Δ+PHO4*, or *pho4Δ* cells (10 mice in each group). Mice infected with the *pho4Δ* mutant survived longer than mice infected with the WT strain (**, *P* = 0.002). (B) Mean CFU from the lungs, brains, and blood from five mice that had succumbed to infection in each group. Compared to mice infected with the WT strain and the *pho4Δ+PHO4* strain, which had similar numbers of lung CFU (*P* = 0.6288), the number of lung CFU of mice infected with the *pho4*Δ mutant was lower than the numbers for mice infected with the WT and *pho4Δ+PHO4* strains (**, *P* = 0.0094; *, *P* = 0.0496). The numbers of brain CFU were similar for WT-strain- and *pho4*Δ+*PHO4* strain-infected mice (*P* = 0.4756) but lower for *pho4*Δ mutant-infected mice (**, *P* = 0.0039). No CFU were obtained from the blood of mice infected with the *pho4Δ* mutant. (C) PAS-stained histological sections of lung and brain samples collected from mice infected with the WT strain and the *pho4Δ* mutant at the time of illness. (D) Survival of mice infected intravenously with 5 × 10^3^ WT, *pho4Δ+PHO4*, and *pho4Δ* yeast cells. The difference in survival between WT-strain- and *pho4*Δ+*PHO4* strain-infected mice and *pho4Δ* mutant*-*infected mice was 25 days (***, *P* < 0.0001). (E) *pho4Δ* CFU were significantly lower in the dissemination model in both the lung (***, *P* < 0.0001) and brain (*, *P* = 0.0195; ***, *P* < 0.0001). (F) PAS-stained sections of lung and brain samples at the time of illness revealed reduced dissemination of *pho4Δ* cells to both the lung and brain compared to WT and *pho4Δ+PHO4* cells. Some cryptococcomas are indicated by black arrowheads. BV, blood vessel. Bars, 100 µm.

To investigate whether cryptococcal Pho4 plays a role in the pathogenesis of cryptococcosis, we compared the virulence of the *pho4Δ* mutant to the virulence of the WT and *pho4Δ+PHO4* strains in a mouse model. As natural cryptococcal infection is acquired by the inhalation of infectious propagules, a mouse inhalation (pulmonary) model was used initially. Mice were infected intranasally with WT, *pho4*Δ+*PHO4*, and *pho4*Δ mutant cells, and their health was monitored daily. The *pho4Δ* mutant was less virulent than the WT and *pho4*Δ+*PHO4* strains (Fig. 3A). The median survival time of mice infected with the *pho4*Δ strain-infected mice was 31 days versus 24 days for both the WT-strain- and *pho4*Δ+*PHO4* strain-infected groups (*P* = 0.002) (Fig. 3A). Fungal burdens were assessed at the time of illness (Fig. 3B) and were found to be similar in the lungs of mice infected with the WT and *pho4*Δ+*PHO4* strains (*P* = 0.6288), but the fungal burden was lower in the lungs of mice infected with the *pho4*Δ mutant strain (*P* = 0.0094). The WT and *pho4*Δ+*PHO4* strains were equally efficient in disseminating to the brain (*P* = 0.4756), and similar numbers of CFU were detected in the blood. In striking contrast, the fungal burden in the brains of mice infected with the *pho4*Δ mutant was minimal (176 CFU/g compared to >3 × 10^6^ CFU/g for the WT and *pho4*Δ+*PHO4* strains; *P* = 0.0039), and no CFU were detected in the blood (Fig. 3B). No brain infection was detected in any of the *pho4*Δ strain-infected mice that survived for 50 days.

Tissue samples collected from all groups of mice that had succumbed to infection were stained with periodic acid-Schiff (PAS) to detect cryptococci (Fig. 3C). Lung infection by the WT strain was typified by the presence of large cryptococcomas containing heavily encapsulated cells that consumed most of the space in the lung, while the *pho4*Δ mutant formed smaller pulmonary cryptococcomas with less capsular material. The WT strain also formed large cryptococcomas in brain sections. In contrast, *pho4*Δ cryptococcomas in brain were difficult to find despite examination of numerous sections from all areas of the brain. This was not surprising given the very low numbers of CFU recovered from *pho4*Δ mutant*-*infected brain tissue.

**FIG 4:**
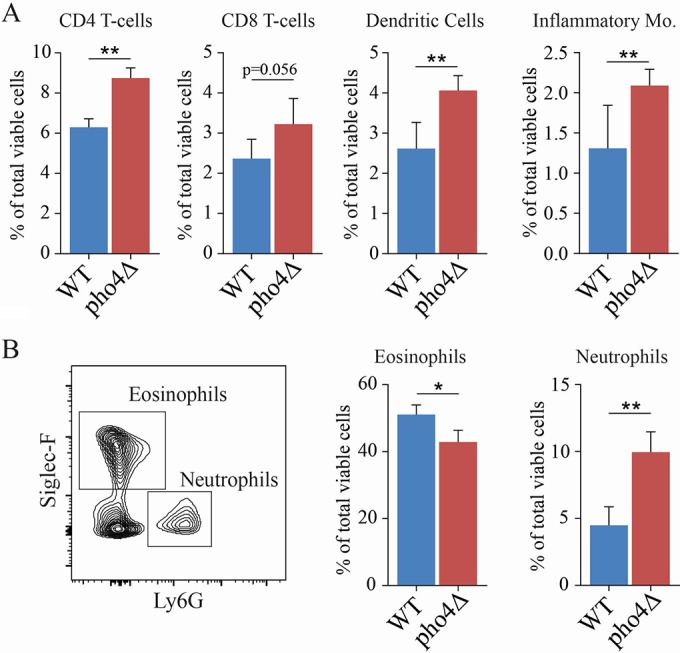
The WT and *pho4*Δ strains trigger a different cellular response in the lungs. Single-cell suspensions of lung cells from mice infected with the WT and the *pho4*Δ mutant were analyzed at 10 days postinfection using flow cytometry. (A) Summary data showing the percentages of CD4^+^ T cells, CD8^+^ T cells, dendritic cells, and Ly6C^hi^ inflammatory monocytes (Mo.). (B) Representative flow cytometry plot showing the gating for eosinophils (Siglec-F^+^) and neutrophils (Ly6G^+^) and summary histograms of their proportions. Data shown are mean percentages plus SDs (*n* = 5). Statistical analysis was performed using Student’s *t* test. Values that are significantly different are indicated by bars and asterisks as follows: *, *P* < 0.05; **, *P* < 0.01.

To determine whether the hypovirulence of the *pho4*Δ mutant coincided with an altered pulmonary immune response, we determined the composition of immune cells in the lungs of mice infected with the WT and *pho4*Δ strains at 10 days postinfection using flow cytometry. The number of total leukocytes isolated from the lungs of WT-strain-infected mice was 3-fold higher than in *pho4Δ* strain-infected mice: 15,240,000 ± 1,442,827 versus 6,713,333 ± 2,788,603 (*P* < 0.01; *n* = 5), respectively. Mean organ burdens were 3.6 × 10^6^ and 6.1 × 10^5^ for WT-strain- and *pho4*Δ strain-infected mice, respectively, and this ~6-fold difference was statistically significant (*P* = 0.0167). Compared to the WT-strain-infected mice, *pho4*Δ strain-infected mice demonstrated significant increases in the proportion of CD4 and CD8 T cells, dendritic cells (CD11c^+^ MHC-II^hi^ [MHC-II stands for major histocompatibility complex class II]) and inflammatory monocytes (CD11b^+^ Ly6G^−^ Ly6C^hi^) (Fig. 4). Interestingly, we noted a modest reduction in the percentage of eosinophils (Siglec-F^+ ^MHC-II^−^) in the lungs of *pho4*Δ strain-infected animals, which correlated with a twofold increase in the proportion of neutrophils (CD11b^+^ Ly6G^+^) (Fig. 4).

### (ii) The virulence of the *pho4*Δ mutant is significantly attenuated in a mouse dissemination model.

Since respiratory inoculation with the *pho4*Δ mutant resulted in minimal dissemination to the brain, we tested whether the apparent block was at the level of the central nervous system or more proximal in a dissemination model. We inoculated 5 × 10^3^ fungal cells directly into the bloodstream via the retro-orbital plexus (Fig. 3D). Mice infected with the WT or *pho4*Δ+*PHO4* strain developed debilitating disease 8 to 10 days postinfection. Strikingly, the *pho4*Δ mutant was even less virulent in this model than in the inhalation model, with all mice infected with the *pho4*Δ mutant surviving at 36 days postinfection (*P* < 0.0001). Fungal burdens in the brain, lungs, and blood were assessed in mice infected with all strains at the time of death (Fig. 3E). Brains harvested from the *pho4*Δ strain-infected mice (day 36) contained an average of 1.1 × 10^7^ CFU/g of brain tissue. This was 31 times less than the average CFU found in the brains of WT-strain- and *pho4*Δ+*PHO4* strain-infected mice at the time of death (8 to 10 days postinfection) (3.47 × 10^8^ and 4.13 × 10^7^ CFU/g of brain tissue, respectively). The numbers of CFU in the lungs of *pho4*Δ mutant-infected mice were also lower at the time of illness. As was observed in the inhalation model, no CFU were detected in blood from *pho4*Δ strain-infected animals.

Lung histology revealed that a significant portion of the lung tissue was healthy in both WT-strain- and *pho4*Δ strain-infected animals, suggesting that death was not due to overwhelming lung infection (Fig. 3F). In contrast, histological examination revealed significant differences in the phenotypes of cryptococcomas in mouse brains. WT cryptococcomas were large, and component cryptococci had large capsules, while with the *pho4*Δ mutant, collections of cryptococci were smaller and less abundant, and individual cells had small capsules.

**FIG 5:**
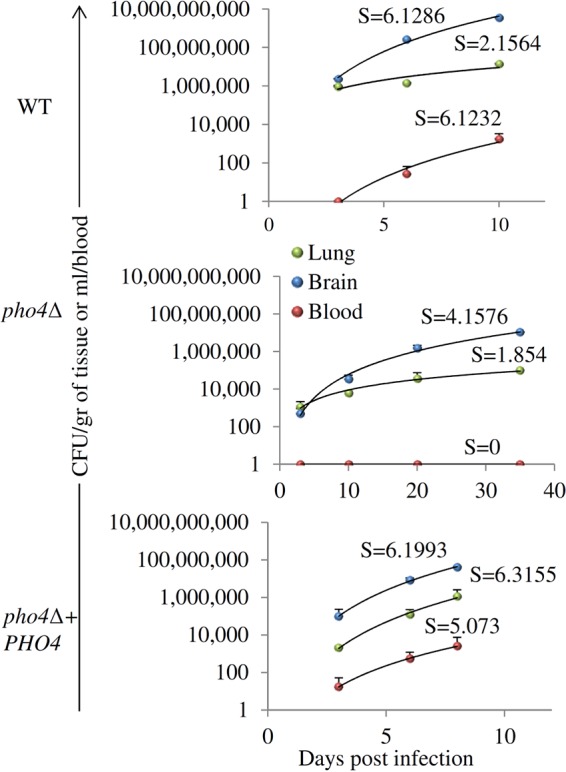
Growth rate of WT, *pho4*Δ, and *pho4Δ+PHO4* strains in tissues and blood as measured in the mouse model of disseminated infection in Fig. 3. The average number of CFU recovered on different days postinfection (day 3, day 6, and the day on which 20% weight loss had been achieved for WT-strain- and *pho4Δ+PHO4* strain*-*infected mice and days 3, 10, 20, and 36 for *pho4*Δ mutant-infected mice) were plotted, and the rate of disease progression was determined in the lung, brain, and blood. Growth is indicated by the regression slope, which is an estimate of the growth rate. The *pho4*Δ growth rate was reduced by 15% to 30% in each organ, and no growth was observed at any time point in blood.

Fungal burdens were measured in lung, brain, and blood at three time points over the course of infection: on days 3, 6, and 9 or 10 for WT-strain- and *pho4*Δ+*PHO4* strain-infected mice and on days 10, 20, and 35 for *pho4*Δ strain*-*infected mice. The results were plotted, and the slope was derived to estimate the rate of growth of each strain (Fig. 5). The growth rates of the WT and *pho4*Δ+*PHO4* strains within each site were similar and 15 to 30% faster than that of the *pho4*Δ mutant. The growth rates of the WT and *pho4*Δ+*PHO4* strains in blood were also similar. In contrast, the *pho4Δ* mutant was never cultured from blood.

### Pho4 is required to tolerate alkaline pH, independent of phosphate availability.

Experiments with mice demonstrated unique infection kinetics of the *pho4*Δ mutant. First, in the inhalation model, which mimics the natural route of infection, dissemination to the brain was minimal. Second, in the intravenous dissemination model, growth of the *pho4Δ* mutant was reduced in both lung and brain. Third, the *pho4*Δ mutant was never cultured from the blood in either model (Fig. 3B and E). None of these features of the infection kinetics was attributable to differences in major virulence phenotypes, as the *pho4*Δ mutant grew at a rate similar to that of the WT at 37°C (and 39°C) and produced WT-like amounts of the classical virulence factors, capsule and melanin, *in vitro* (Fig. S6). This is in agreement with phenotypic data obtained for the *hlh3Δ* mutant during systematic functional profiling of transcription factor networks in *C. neoformans* (10). However, capsule formation in the *pho4*Δ mutant was reduced during lung and brain infection *in vivo* (Fig. 3C and F).

10.1128/mSphere.00381-16.8 FIG S6  The *pho4*Δ mutant grows in a manner similar to the WT at elevated temperature and produces WT-like capsule and melanin. (A) Spot dilution assays were performed on YPD or, for melanin production test, on MM supplemented with 29.4 mM KH_2_PO_4_ and 1 mM l-3,4-dihydroxyphenylalanine (l-DOPA). (B) For capsule induction, WT and *pho4*Δ cells were grown on MM supplemented with KH_2_PO_4_. Capsules were visualized using negative staining with Indian ink. Download FIG S6, PDF file, 1.1 MB. Copyright © 2017 Lev et al. 2017 Lev et al. This content is distributed under the terms of the Creative Commons Attribution 4.0 International license.

**FIG 6:**
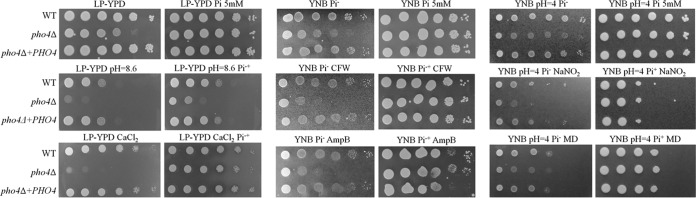
Role of Pho4 in stress tolerance in the presence and absence of phosphate. All spot dilution assays were performed on low-phosphate medium (LP-YPD, YNB Pi^−^, or YNB Pi^−^ [pH 4]) with and without 5 mM phosphate. The plates also contained stress agent 0.5 M CaCl_2_, calcofluor white (CFW) (0.5 mg/ml), amphotericin B (AmpB) (0.6 µg/ml), NaNO_2_ (1.25 mM), or menadione (MD) (10 µM), or the pH was adjusted to 8.6, as indicated. Alkaline pH sensitivity is the only *pho4*Δ phenotype not restored by phosphate supplementation.

It was recently reported that Pho4 from *C. albicans* plays a role in stress tolerance (13, 15), while Pho4 from *S. cerevisiae* affects stress tolerance in a P_i_-dependent and P_i_-independent manner (16). We therefore tested tolerance of the cryptococcal *pho4*Δ mutant to a variety of stresses in the presence and absence of phosphate. We used low-phosphate medium (low-phosphate YPD [LP-YPD], YNB Pi^−^, or YNB Pi^−^ [pH 4] [see Materials and Methods]) with and without 5 mM phosphate to differentiate between phosphate-dependent and -independent functions of Pho4. In the absence of phosphate, the *pho4*Δ mutant exhibited sensitivity to calcofluor white, amphotericin B, a high calcium concentration, nitrosative stress, and alkaline pH (Fig. 6). The tolerance of the *pho4*Δ mutant to oxidative stress induced by hydrogen peroxide, methylglyoxal, and menadione was similar to that of the WT (Fig. 6 and data not shown). With the exception of sensitivity to alkaline pH, all phenotypic defects were rescued by phosphate supplementation. Thus, in contrast to the role of Pho4 in maintaining cell wall integrity and cation and antifungal drug tolerance in the absence of phosphate, its role in alkaline pH tolerance was independent of phosphate availability.

**FIG 7:**
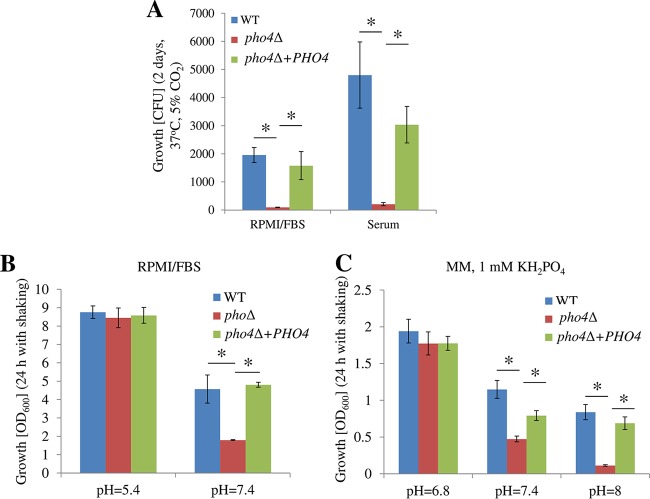
Pho4 is required for optimal growth of *C. neoformans* at physiological and alkaline pH**.** (A) The strains (100 μl; 1,000 cells/ml) were grown in standard tissue culture medium RPMI-FBS or murine serum as a stationary culture for 2 days at 37°C and 5% CO_2_. Growth was measured by counting CFU. (B and C) Fungal cells were grown in shaking cultures at 30°C in RPMI-FBS buffered at pH 5.4 and 7.4 (B) or in MM containing 1 mM KH_2_PO_4_ with the pH adjusted to 6.8, 7.4, and 8 (C). Growth was initiated from a starting OD_600_ of 0.05 and quantified 24 h later by measuring OD_600_. Data shown are means ± SDs (three biological replicates). Statistical analysis was performed using *t* test. *, *P* < 0.05.

We considered whether the sensitivity of the *pho4*Δ mutant to alkaline pH in tissues and blood would explain the reduced growth and negative cultures, respectively, in mice. Growth of the *pho4*Δ mutant was compared with that of the WT and *pho4*Δ+*PHO4* strains in mouse serum (pH 7.4) and standard cell culture medium (RPMI-FBS [see Materials and Methods] at pH 7.4, containing ~5 mM phosphate) at 37°C in a 5% CO_2_ atmosphere. Consistent with the experiments *in vivo*, growth of the *pho4*Δ mutant was severely compromised in both media compared to the WT and reconstituted strains (Fig. 7A). To investigate whether alkaline pH is responsible for the growth defect, growth was also assessed in RPMI-FBS with the pH buffered to pH 5.4 or 7.4 (Fig. 7B), and minimal medium (MM) at pH 6.8, 7.4, and 8 (Fig. 7C). The MM was supplemented with 1 mM KH_2_PO_4_, which is 10-fold higher that the concentration of P_i_ required to suppress PHO pathway activation at pH 5.6, as established using extracellular acid phosphatase activity as a readout (Fig. S7). In both MM and RPMI-FBS, the *pho4*Δ mutant grew more slowly than the WT and *pho4*Δ+*PHO4* strains when the pH was higher than 7, while all three strains grew similarly when the pH was lower than 7. Collectively, these results demonstrate that alkaline pH, rather than serum or other components in RPMI 1640 medium, contributes to the *pho4*Δ growth defect.

10.1128/mSphere.00381-16.9 FIG S7  Extracellular acid phosphatase (APase) activity of *C. neoformans* grown in minimal medium supplemented with different concentrations of KH_2_PO_4_. Aph1 activity, as determined by measuring the hydrolysis of pNPP at 420 nm, is shut down at a KH_2_PO_4_ concentration of 100 µM. Download FIG S7, PDF file, 0.01 MB. Copyright © 2017 Lev et al. 2017 Lev et al. This content is distributed under the terms of the Creative Commons Attribution 4.0 International license.

### Pho4 is required for induction of phosphate transporters and phosphate uptake under physiological conditions/alkaline pH when phosphate is available.

The high-affinity phosphate transporters Pho84 and Pho840 are H^+^/P_i_ symporters and therefore function more efficiently at acidic pH. To determine whether phosphate acquisition by WT *C. neoformans* is indeed compromised at alkaline pH, the uptake of radioactive orthophosphate by phosphate-deprived cells at acidic pH (5.4) and physiological pH (7.4) was assessed. Phosphate uptake was reduced at pH 7.4 compared with pH 5.4, over a 150-min time course (Fig. S8). In this experiment, the induction of phosphate transporters was maximized by prior phosphate starvation. Thus, our results confirm that phosphate uptake is reduced under conditions of alkaline pH.

10.1128/mSphere.00381-16.10 FIG S8  Uptake of radioactive orthophosphate (P^32^) by WT *C. neoformans* is affected by pH**.** Fungal cells were preincubated in phosphate-free medium (MM pH 5.4 or MM pH 7.4) for 2 h. Samples were collected at different time points following the addition of KH_2_PO_4_ (100 µM) and ^32^P and analyzed by scintillation counting. Download FIG S8, PDF file, 0.01 MB. Copyright © 2017 Lev et al. 2017 Lev et al. This content is distributed under the terms of the Creative Commons Attribution 4.0 International license.

**FIG 8:**
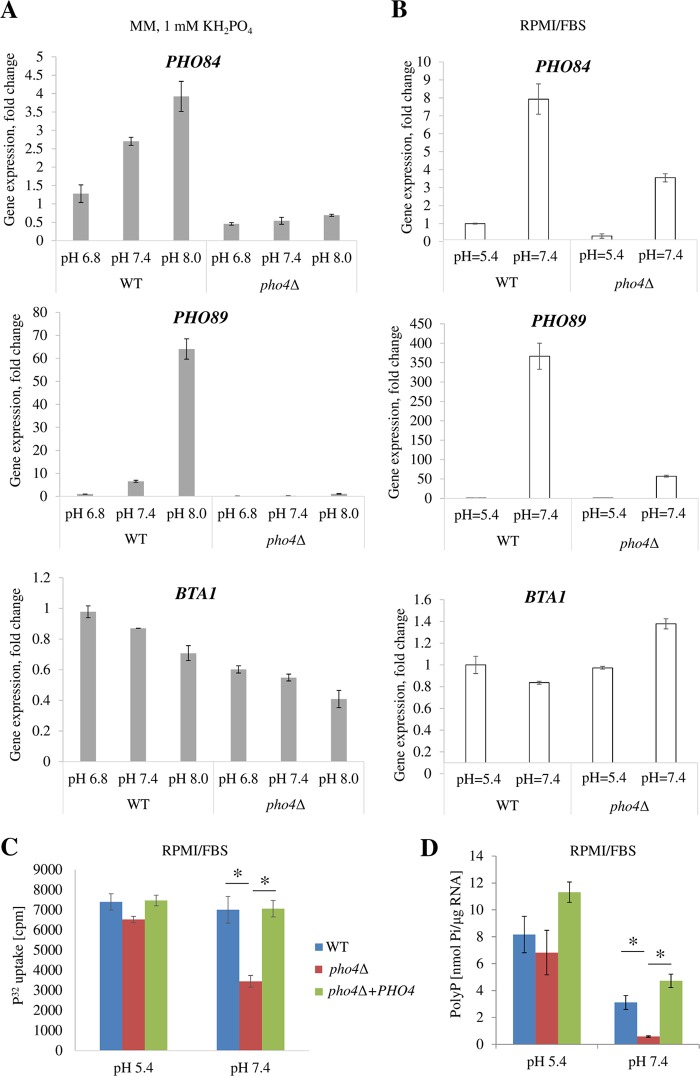
Pho4 is required to maintain growth of *C. neoformans* at alkaline pH by upregulating expression of phosphate transporters and increasing phosphate uptake. (A and B) The indicated strains were grown in MM containing 1 mM KH_2_PO_4_ with the pH adjusted to 6.8, 7.4, and 8 or in RPMI-FBS at pH 5.4 and 7.4. Growth was initiated from a starting OD_600_ of 1 for 4 to 5 h, and the expression of the indicated Pho4-dependent genes was quantified by qPCR using *ACT1* expression for normalization. (C) Uptake of ^32^P orthophosphate by the cultures grown for 5 h in RMPI-FBS at pH 5.4 and 7.4 from the starting OD_600_ of 1. (D) PolyP contents of the cultures grown for 24 h in RMPI-FBS at pH 5.4 and 7.4 from a starting OD_600_ of 0.05. PolyPs were quantified using a malachite green colorimetric assay. The results represent the means ± SDs. Statistical analysis was performed using *t* test. *, *P* < 0.05.

To determine whether a phosphate starvation response is activated at alkaline pH when phosphate is available, we compared the induction of representative Pho4 effector genes involved in phosphate mobilization (*BTA1*) and uptake (*PHO84* and *PHO89*) in the WT and *pho4Δ* strains following growth in MM supplemented with 1 mM KH_2_PO_4_ at pH 6.8, 7.4, and 8 and RPMI medium at pH 7.4 or pH 5.4. The concentration of phosphate in RPMI (~5 mM) is 50 times higher than that required to repress activation of the PHO pathway in the WT at pH 5.6 (~100 µM [Fig. S7]). In MM containing 1 mM KH_2_PO_4_, expression of both phosphate transporters increased in the WT, but not in the *pho4*Δ mutant, as the pH increased (Fig. 8A). In contrast, expression of *BTA1* in both the WT and *pho4*Δ mutant strains did not increase with increasing pH (Fig. 8A). A similar trend was observed in RPMI culture medium except that some induction of phosphate transporters was observed in the *pho4*Δ mutant at pH 7.4, although less than in the WT. Importantly, compared with the WT, the reduced expression of phosphate transporters in the *pho4*Δ mutant in RPMI (pH 7.4) correlated with reduced uptake of radioactive phosphate over a 5-h period in the same medium (Fig. 8C) and lower levels of PolyPs (measured after 24 h of incubation [Fig. 8D]). The expression of *BTA1*, *APH4*, and *VTC4* in WT and *pho4*Δ cells was not induced in RPMI at pH 7.4 (Fig. 8A and data not shown).

Collectively, these findings suggest that fungal cells experience phosphate starvation under physiological conditions, due to diminished efficiency of P_i_/H^+^ symporters that require protons for P_i_ uptake and are thus adapted to functioning at acidic pH. Activation of the PHO pathway in the WT, which is abrogated in the *pho4*Δ mutant, can overcome phosphate deficiency by upregulating phosphate transporters, especially the Na^+^/P_i_ cotransporter Pho89, which functions optimally at basic pH (17). Consequently, *pho4*Δ growth and stress tolerance are compromised at alkaline pH.

### Peripheral blood monocytes arrest *pho4Δ* growth.

**FIG 9:**
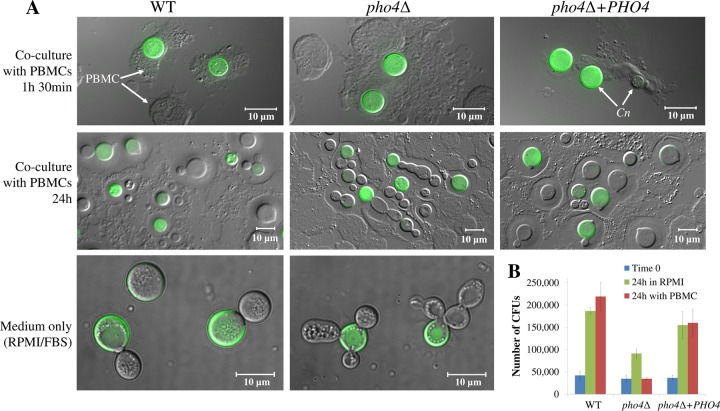
Growth of the *pho4*Δ mutant is delayed in physiological medium and is further inhibited by the presence of PBMCs. (A) Uptake (1.5 h) and proliferation (24 h) of FITC-labeled fungal cells (green) in human PBMCs. RPMI-FBS served as a control. The images were produced by overlaying differential interference contrast micrograph (gray) and green fluorescence. Arrows indicate labeled and unlabeled (daughter) fungal cells (*C. neoformans* [*Cn*]) and PBMCs. Note that *pho4*Δ cells exhibit a cell separation defect following incubation with PBMCs and in RPMI-FBS alone. (B) Proliferation of the fungal cells in the presence and absence of PBMCs. Time zero refers to the number of CFU in the beginning of coincubation of the fungal cells with PBMCs. To quantify fungal cells, monocytes were lysed by adding 0.05% SDS, and fungal cells were serially diluted and plated. The results represent the means ± SDs (three biological replicates).

Blood cultures were consistently negative in mice infected with the *pho4*Δ mutant (Fig. 3B and E). It has been shown in zebrafish models and using live-cell imaging of mouse brain vasculature, that circulating monocytes phagocytose WT *C. neoformans*, allow fungal replication (18–20), and provide a vehicle for fungal dissemination to the brain via the “Trojan horse” mechanism (21–23). Given that we observed a nitrosative stress tolerance defect in the *pho4*Δ mutant, we compared the extent of proliferation of opsonized fluorescence-labeled WT, *pho4*Δ+*PHO4*, and *pho4Δ* strains following culture with peripheral blood mononuclear cells (PBMCs). Ingestion/binding by PBMCs after 1.5 and 24 h of coculture was visualized by fluorescence microscopy (Fig. 9A), while the viability/extent of cryptococcal proliferation before (time zero) and after 24 h of incubation was determined by quantitative culture (Fig. 9B). Following 1.5 h of coculture with PBMCs, ~75% of fungal cells of each strain were associated with or taken up by PBMCs, as indicated by flow cytometric analysis (data not shown). Fluorescence microscopy confirmed that most cryptococcal cells had been phagocytosed by PBMCs (Fig. 9A).

By 24 h, all fungal strains proliferated, as indicated by the presence of unlabeled daughter cells. Interestingly, the *pho4*Δ mutant exhibited a cell separation defect in coculture at 24 h (Fig. 9A). However, this defect was also evident in RPMI medium alone (Fig. 9A). Quantitative culture revealed similar extents of proliferation of the WT and *pho4Δ+PHO4* strains after 24-h incubation in the presence and absence of PBMCs (Fig. 9B). In contrast, the number of* pho4*Δ CFU obtained at 24-h coculture was the same as at time zero, suggesting that many of the fungal cells in Fig. 9A are not viable, despite the evidence of cell division. The growth of the *pho4*Δ mutant in medium alone was more than twofold greater than that in coculture, but less than that of the WT and *pho4*Δ+*PHO4* strains (*P* = 0.05). Our results show that the probability of live *pho4*Δ cells being transported to the brain in monocytes is low, consistent with the low level of brain infection we observed in our murine models.

## DISCUSSION

*C. neoformans* possesses an evolutionarily divergent set of transcription factors that share little homology with their counterparts in nonpathogenic model fungi (10). Using a transcription factor knockout library constructed by Jung et al. (10), we identified the Pho4 ortholog in the serotype A strain H99 and established that Pho4 is essential for the cryptococcal response to phosphate starvation. By tagging Pho4 with a red fluorophore, we confirmed that Pho4 is responsive to phosphate by observing it shuttle between the nucleus and cytoplasm in response to phosphate availability. Similar to other yeasts, *C. neoformans* Pho4 is likely to be regulated by the cyclin-dependent kinase inhibitor Pho81 and CDK-cyclin complex Pho85-Pho80. Given that Toh-e et al. established that the CDK-encoding *PHO85* gene is essential for the viability of *C. neoformans* serotype D (7), we utilized the commercially available CDK inhibitor, purvalanol A, to investigate the impact of Pho85 inhibition on PHO pathway activation. Purvalanol A treatment uncoupled PHO signaling from phosphate sensing, leading to constitutive activation of the pathway irrespective of phosphate availability and establishing an epistatic relationship between Pho85 and Pho4 (Fig. 2A). Our experiments and those of Toh-e et al. (7) with serotype D are consistent with activation of Pho4 via Pho81-mediated inhibition of CDK Pho85 during phosphate deprivation.

We also showed for the first time that *pho4*Δ growth in P_i_-deficient medium was markedly reduced, indicating that Pho4 is essential for facilitating the mobilization of P_i_ from dispensable phosphorylated compounds found intracellularly. This is supported by the fact that Pho4 triggers induction of the intracellular acid phosphatases Aph3 and Aph4 and the vacuolar and extracellular acid phosphatase Aph1, which utilizes a variety of organic compounds as the substrate, i.e., amino acids, lipids, and sugars (9). Pho4 also promotes P_i_ uptake from the extracellular milieu, since *pho4*Δ growth was markedly delayed when β-glycerol phosphate was provided as the sole source of phosphate. This was in contrast to the robust growth of the WT. We showed previously that β-glycerol phosphate is a substrate of the sole secreted acid phosphatase in *C. neoformans*, Aph1 (9). Pho4-dependent expression of *APH1* and of the genes encoding the high-affinity P_i_ transporters, *PHO89*, *PHO84*, and *PHO840*, facilitates the release of P_i_ from complex sources and the transport of free P_i_ across the plasma membrane, respectively.

### Alkaline stress mimics phosphate deprivation, leading to *pho4*Δ stress hypersensitivity.

We observed that phosphate supplementation promotes tolerance of the *pho4*Δ mutant to amphotericin B, cell wall stress, high cation concentration, and nitrosative stress (Fig. 6). It is important to note that all the stress tests were performed in standard acidic growth media. The tolerance of the *pho4*Δ mutant to these stresses in the presence of phosphate suggests that phosphate uptake by the mutant is sufficient to overcome the stress. This is consistent with our observation that the *pho4*Δ mutant takes up radioactive orthophosphate and accumulates polyPs to extents similar to those of the WT strain when both strains are grown in YPD medium (~2.5 mM free phosphate). Our findings are in contrast to those reported for the *pho4*Δ mutant of *C. albicans*, which was sensitive to menadione-induced oxidative stress and accumulated less polyPs even in the presence of phosphate (13).

Unlike sensitivity to other stresses, alkaline pH sensitivity of the *pho4*Δ mutant was not remediated by the addition of phosphate. Similar to *C. neoformans*, Pho4 is essential for alkaline pH tolerance in *C. albicans* and *S. cerevisiae* (24, 25). In these yeasts, alkaline pH triggers rapid degradation of polyP stores, and in *S. cerevisiae*, the induction of Pho4 effector genes, including the acid phosphatase-encoding gene *PHO12* and components of the Vtc complex (13, 26, 27). Exposure to alkaline pH is thought to mimic phosphate deprivation, as phosphate uptake by fungal cells via P_i_/H^+^ symporters is less efficient under these conditions (28; reviewed in reference 27). Indeed, we confirmed that this is the case in *C. neoformans* by demonstrating reduced uptake of radioactive orthophosphate by P_i_-starved cryptococci at pH 7.4 compared to pH 5.4 (Fig. S8) and that phosphate uptake at pH 7.4 was reduced even further in the absence of Pho4 (Fig. 8C). PolyP reservoirs were also reduced in the WT, and to an even greater extent in the *pho4Δ* mutant at pH 7.4 (Fig. 8D). These observations suggest that the *pho4*Δ mutant is P_i_ deprived at physiological pH even in the presence of phosphate. The vulnerability of the *pho4*Δ mutant to host-derived stress, including nitrosative stress, is therefore likely to be increased during infection.

### Alkaline stress leads to activation of a subset of Pho4-responsive genes.

Transport of P_i_ under alkaline pH conditions is likely to be mediated predominantly by the Na^+^/P_i_ cotransporter Pho89, which does not require protons to transport P_i_ across the plasma membrane. This is supported by the fact that *PHO89* gene expression in the WT was upregulated 366-fold in RPMI at pH 7.4 compared to pH 5.4 (Fig. 8A). Expression of *PHO89* was also increased, albeit to a lesser extent of ~57-fold, in the *pho4*Δ mutant under physiological conditions (Fig. 8A). This suggests the presence of a Pho4-independent phosphate response mechanism involved in maintaining phosphate uptake during host infection. In future experiments, it will be interesting to see whether the defects in the *pho4*Δ mutant can be reversed by the overexpression of *PHO89*. In *C. neoformans*, *PHO89* is also a target of the alkaline-response transcription factor Rim101 (29). Activation of Rim101 is therefore likely to contribute to the induction of *PHO89* in the *pho4*Δ mutant at alkaline pH. Interestingly, comparison of the Pho4 (7) and Rim101 (29) effector genes revealed very limited overlap, suggesting that these pathways regulate alkaline pH tolerance independently of each other. This conclusion is supported by the finding that, in contrast to the *pho4*Δ mutant, the *rim101*Δ mutant grew similarly to the WT at physiological pH and that the *rim101*Δ mutant is hypervirulent rather than hypovirulent (29).

Surprisingly, P_i_ transporter-encoding genes, but not Pho4 effector genes involved in phosphate mobilization (*VTC4*, *BTA1*, and *APH4*), were upregulated at alkaline pH. Furthermore, upregulation of the transporters at alkaline pH was modest in comparison to their induction in the absence of phosphate: in RPMI at pH 7.4 versus pH 5.4, *PHO84* and *PHO89* were upregulated 8-fold and 366-fold, respectively, while in response to phosphate starvation (MM-KCl versus MM-KH_2_PO_4_), these genes were upregulated 84-fold and 4,096-fold, respectively. Moderate activation of only a subset of Pho4 effector genes, including, but not limited to, P_i_ transport in response to alkaline pH may therefore be sufficient to alleviate P_i_ deficiency and achieve normal cellular function. Notably, in *S. cerevisiae*, low phosphate induces partial Pho4 phosphorylation and differential induction of *PHO84* and *PHO5* (the *APH1* ortholog) (30).

Taken together, these findings suggest that activation of only a subset of Pho4-responsive genes at alkaline pH, including phosphate transporters, is sufficient to allow *C. neoformans* to adapt to growth at alkaline pH.

### Phenotypic comparison of the *pho4*Δ mutant and the phosphate transporter mutant.

The effect of compromised induction of P_i_ transporters in the *pho4*Δ mutant is largely phenocopied in the P_i_ transporter-deficient *pho84*Δ *pho840*Δ *pho89*Δ mutant (8). The similarities include sensitivity to alkaline pH, inability to grow in cell culture medium, hypovirulence in the mouse inhalational model, and sensitivity to killing by macrophages. Furthermore, Kretschmer et al. (8) identified a cell division defect in the *pho84*Δ *pho840*Δ *pho89*Δ mutant, which is reminiscent of the *pho4*Δ phenotype under physiological conditions (Fig. 9A). Despite these similarities, some differences were also observed: unlike the *pho84*Δ *pho840*Δ *pho89*Δ mutant, the *pho4*Δ mutant produced WT levels of polyPs, capsule, and melanin under standard growth conditions *in vitro* (although the *pho4*Δ mutant did produce smaller capsules during murine infection). Furthermore, Kretschmer et al. showed that the *pho84*Δ *pho840*Δ *pho89*Δ mutant was less virulent in an intranasal model of infection (8), compared to the *pho4*Δ mutant in our experiments (Fig. 3A), but in contrast to the *pho4*Δ mutant, the *pho84*Δ *pho840*Δ *pho89*Δ mutant exhibited significant dissemination to the brain (8). Some of these differences could be attributed to the use of a different mouse strain and different inoculation doses (8). The difference in the rates of dissemination of the *pho84*Δ *pho840*Δ *pho89*Δ and *pho4*Δ mutants could also be due to decreased expression of additional Pho4 effector genes, such as proton-dependent nutrient transporters (amino acids, calcium, and iron) as reported by Toh-e et al. (7). These transporters are likely to contribute to cryptococcal tolerance to physiological pH and proliferation in the host. The virulence of the *pho84*Δ *pho840*Δ *pho89*Δ mutant and its ability to infect the brain in a dissemination model was not investigated in the study of Kretschmer et al. (8).

### Physiological pH stress sensitivity, particularly in blood, leads to a *pho4*Δ dissemination defect.

Our studies with a murine inhalation model demonstrate that several factors contribute to the failure of the *pho4*Δ mutant to cause brain infection: slower growth in the lungs leading to lower lung fungal burdens and lower rates of pathogen release into the blood, lack of proliferation or survival in the alkaline pH of the blood, and sensitivity to macrophage-induced stress. *pho4Δ* lung infection coincided with less eosinophilia and a higher proportion of neutrophil infiltration, which is consistent with the reduced fungal burdens and healthier lung pathology observed. Although neutrophils kill *C. neoformans* more efficiently than monocytes do (31, 32), their protective role in confining cryptococcosis to, or clearing it from, the lung remains controversial (for a review, see reference 33). However, in our case, it cannot be ruled out that increased proportions of neutrophils, coupled with less eosinophil-induced tissue damage, contributed to decreased *pho4Δ* lung burdens and reduced dissemination. The altered immune response toward the *pho4Δ* mutant could be due to its inability to activate the PHO pathway even though such activation in the WT is expected to be mild due to the predominantly more acidic environment within a cryptococcoma. Even mild activation of the PHO pathway in the WT, but not in the *pho4*Δ mutant, is likely to result in a different surface topology. Genome-wide analysis of P_i_-responsive genes in *C. neoformans* serotype D showed that, in addition to P_i_ transporters, Pho4 regulates the expression of numerous other genes, including those encoding amino acid, calcium, and siderophore transporters (7).

We also observed that the *pho4*Δ mutant had a cell separation defect when grown under physiological conditions, which resulted in a reduced number of CFU. The rate of* pho4*Δ proliferation, as measured by the number of CFU, was reduced even further in the presence of PBMCs. This may be attributed to the fact that the *pho4*Δ mutant is sensitive to nitrosative stress in the absence of phosphate. As we have confirmed in this study that the *pho4*Δ mutant experiences phosphate starvation at alkaline pH due to phosphate transporter inefficiency, it is likely that the *pho4*Δ mutant will also be sensitive to macrophage-induced nitrosative stress during infection. Similar to our findings, proliferation of the *pho4*Δ mutant of *Candida albicans* in a lung macrophage cell line was also compromised (13). However, the cause of increased vulnerability of *C. albicans* and *C. neoformans pho4*Δ mutants to phagocytes appears to be different: *C. albicans* Pho4 was implicated in the regulation of metal homeostasis and therefore in the functionality of Cu/Zn-requiring superoxide dismutases and superoxide resistance (13). Unlike the *C. albicans pho4*Δ mutant, the *C. neoformans* *pho4*Δ mutant was not sensitive to menadione-induced oxidative stress but rather was hypersensitive to nitrosative stress.

The *pho4Δ* mutant was also markedly less virulent than the WT in the cryptococcal dissemination model (infection via intravenous inoculation) and grew more slowly than the WT in lung and brain. Unlike the WT, no CFU were recovered from blood sampled from mice infected with the *pho4*Δ mutant at any stage of infection in either the intranasal or dissemination models. The role of Pho4 in the virulence of *C. albicans* has also been investigated in a mouse dissemination model, as well as a gut model (13, 34, 35). In the mostly acidic environment of the gut, colonization by the WT and the *pho4*Δ mutant was similar (13, 34, 35), while in the dissemination model, the *pho4*Δ mutant was hypovirulent, albeit the loss of virulence was not as dramatic as observed for the *C. neoformans pho4*Δ mutant (13, 34, 35). These observations suggest that alkaline pH tolerance regulation is a common feature of Pho4 in pathogenic fungi, contributing to disseminated disease.

Differences in the rate of growth of the *pho4*Δ mutant in the tissues and blood could be partly explained by the fact that fungal cells are clustered in the organs and dispersed by the circulation system. Within the tissues, fungal cells proliferate in close proximity, forming cryptococcomas. It has been shown that the pH within the microenvironment of the cryptococcoma is acidic (pH ~5.5) due to the production of organic acids like acetic acid (6, 36). In contrast, cryptococci that have been released into the bloodstream are dispersed in the circulation system until they reach the vasculature. Cryptococci in blood are therefore more exposed to the detrimental impact of alkaline (physiological) pH on growth and nutrient uptake, as they are less able to influence the pH of their microenvironment. Hence, they exhibit abrogated growth and a cell separation defect and become more susceptible to killing by monocytes. The ability of the *pho4*Δ mutant strain to form cryptococcomas in the lung following intranasal infection may therefore be the reason why the mutant is more virulent in this model than in the dissemination model where a combination of low seeding density and dispersal by the circulation system does not allow infection to establish as readily as in the tissues.

In summary, we have demonstrated that Pho4 is an important facilitator of cryptococcal dissemination to the host brain. Specifically, Pho4 enables *C. neoformans* to acquire sufficient phosphate at physiological pH, promoting growth and stress tolerance during host infection, especially in blood.

## MATERIALS AND METHODS

### Fungal strains, growth media, and plasmids.

Wild-type *C. neoformans* var. *grubii* strain H99 (serotype A, *MAT*α), a gift from John Perfect, Duke University Medical Center, was used in this study. Routinely, the cells were grown on Sabouraud dextrose agar (SDA) or YPD broth (1% yeast extract, 2% peptone, and 2% dextrose). For phenotypic assays, YNB (yeast nitrogen base) without phosphate (catalog no. 114027812; MP Biomedicals) was used, supplemented with glucose (0.5% [wt/vol]) and NaCl (0.01%) (YNB Pi^−^). Acidic YNB for the nitrosative stress tolerance test was prepared by adding 25 mM sodium succinate (pH 4) to the YNB Pi^−^. Some of the phenotypic assays used low-phosphate YPD (LP-YPD) prepared as described in reference 37. YNB and LP-YPD agar plates were supplemented with stress-inducing agents as indicated. Minimal medium (MM) (15 mM glucose, 10 mM MgSO_4 _⋅ 7H_2_O, 13 mM glycine, 3 µM thiamine-HCl) was used as a base for multiple derivative media. For phenotypic testing, MM was supplemented with 29.4 mM KCl (MM-KCl) or with 29.4 mM KH_2_PO_4_ (MM-KH_2_PO_4_) or with 29.4 mM β-glycerol phosphate. For the experiments requiring different pHs, MM was buffered with morpholineethanesulfonic acid (MES) (pH 5.4), or HEPES (pH 6.8, 7.4, or 8) at 20 to 100 mM. Buffered RPMI-FBS (RPMI medium) was prepared by combining RPMI 1640 medium powder (Sigma), glutamine, fetal bovine serum (FBS) (10%), MES (100 mM for pH 5.4), or HEPES (100 mM for pH 7.4).

### Screening for transcription factors involved in phosphate homeostasis in *C. neoformans.*

All transcription factor knockout library strains (10) were grown overnight on SDA agar, and a small amount of cells was inoculated in 100 µl of inducing medium (MM-KCl) in a 96-well plate. The cells were incubated for 3 h at 30°C with mild agitation using a plate shaker. Sodium acetate (pH 5.2) and *p*-nitrophenyl phosphate (pNPP) were then added to a final concentration of 50 mM and 2.5 mM, respectively. The plates were incubated at 37°C for 5 min, and the reactions were stopped by the addition of saturated Na_2_CO_3_ (50 µl). Extracellular acid phosphatase activity was indicated by the development of a yellow color due to the hydrolysis of pNPP (colorless) to pNP (yellow).

### Spot dilution assay.

*C. neoformans* strains were grown overnight at 30°C in YPD broth. The cells were pelleted by centrifugation, washed, and resuspended at a concentration of 10^6^ cells per 5 µl. Serial 10-fold dilutions were prepared, and 5-µl samples of each suspension were spotted onto the various agar media described in “Fungal strains, growth media, and plasmids” above. Macroscopic growth was recorded after 2 or 3 days of growth at 30°C.

### Fungal RNA extraction and quantitative PCR.

Fungal cells were pelleted by centrifugation and snap-frozen in liquid nitrogen. The cells were homogenized by bead beating in the presence of glass beads (425 µm to 600 µm) and TRIzol (Ambion), and RNA was extracted following the manufacturer’s instructions. Residual DNA in RNA samples was removed by treatment with RQ DNase (Promega). cDNA was synthesized using Moloney murine leukemia virus reverse transcriptase (Promega). Expression of PHO genes (listed in Table 1) was quantified by quantitative PCR (qPCR) on a Rotor-Gene 6000 (Corbett Research) using Platinum SYBR green qPCR SuperMix-UDG (Life Technologies, Inc.). Primers used for qPCR are listed in Table S1 in the supplemental material. The expression of each gene was quantified using the 2^−ΔΔ*CT*^ method, where actin (*ACT1* [CNAG_00483]) served as a reference (housekeeping) gene.

10.1128/mSphere.00381-16.2 TABLE S1  Primers used in this study. Download TABLE S1, DOCX file, 0.01 MB. Copyright © 2017 Lev et al. 2017 Lev et al. This content is distributed under the terms of the Creative Commons Attribution 4.0 International license.

10.1128/mSphere.00381-16.1 TEXT S1  Supplementary methods. Download TEXT S1, DOCX file, 0.02 MB. Copyright © 2017 Lev et al. 2017 Lev et al. This content is distributed under the terms of the Creative Commons Attribution 4.0 International license.

### Construction of Pho4-mCherry and *pho4*Δ+*PHO4* strains.

A WT *C. neoformans* strain expressing *HLH3/PHO4* constitutively as an mCherry fusion protein under the control of the glycerol-3-phosphate dehydrogenase promoter (GPD1p) was constructed in two steps. In the first step, a PHO4-mCherry-NEO gene fusion was constructed by using pNEO-mCherryht vector and was integrated into the *C. neoformans* genome (Fig. S5A and B). The 3′ end of the *PHO4* coding region (CNAG_06751) without the stop codon (positions 1384 to 2495) was PCR amplified from WT H99 genomic DNA using the primers HLH3-5’XhoI-s and HLH3-3’-NotI-a. The resulting fragment was ligated into the XhoI and NotI sites of pNEO-mCherryht, joining it in frame with the mCherry sequence. A 1,153-bp portion of the genomic sequence downstream of *PHO4* was also PCR amplified from WT genomic DNA using the primers 3’HLH3-SacI-5’s and 3’HLH3-KpnI-3’a, and ligated into the SacI and KpnI sites of mCherry-NEO downstream of *NEO*. The resulting plasmid, pPHO4-mCherry-NEO (Fig. S5A), was linearized with KpnI and introduced into the genome of the WT H99 strain using biolistic transformation (Fig. S5B) by the method of Toffaletti et al. (38). Geneticin-resistant colonies were screened by PCR amplification across the PHO4-mCherry-NEO recombination junctions (Fig. S5D). The successful transformant was used in the next step for introduction of the GPD1 promoter. All primers used are listed in Table S1.

In the second step, the *GPD1* promoter was fused to *PHO4*-mCherry (Fig. S5C). This step involved the PCR amplification of four overlapping PCR-generated fragments. These fragments included the following: (i) 1,152 bp of genomic DNA upstream of the *PHO4* coding sequence, (ii) the hygromycin B resistance (Hyg^r^) cassette (39), (iii) the *GPD1* promoter (GPD1p) to drive expression of Pho4-mCherry, and (iv) part of the *PHO4* coding sequence (positions 1 to 975). *PHO4* genomic sequence was PCR amplified from WT H99 genomic DNA using the primer pair HLH3-Nest-5’s and HLH3p-NEO-a. Hyg^r^ was generated using the primer pair Neo-s and HygB-a (39). GPD1p was PCR amplified from *C. neoformans* genomic DNA using primer pair HYG-GPD1p-s and GPD1p-a. The 5′ end of the *PHO4* coding sequence was PCR amplified from WT H99 genomic DNA using the primer pair GPD1p-HLH3-s and HLH3c-int-a. The final overlap PCR was performed using the primer pair HLH3-Nest-5’s and HLH3c-int-a. The final construct was used to transform PHO4-mCherry-Neo transformants (Fig. S5C) using biolistic transformation. Hygromycin-resistant colonies were screened by PCR amplifying regions across the GPD1p-PHO4-HYG recombination junctions (Fig. S5E and F). All primers used are listed in Table S1.

To construct the *PHO4* reconstituted strain (*pho4Δ+PHO4* strain), the genomic *PHO4* locus, including the coding region flanked by 1,278 bp of sequence upstream and 740 bp downstream, was PCR amplified from WT H99 genomic DNA using the primer pair HLH3-5’s and HLH3t-a. The neomycin resistance (Neo^r^) cassette was PCR amplified from pJAF1 (40) using the primer pair (HLH3t)-Neo-s and Neo-a. The two fragments were then fused together by overlap PCR using the primer pair HLH3-5’s and Neo-a, and the construct was introduced into the *pho4*Δ mutant obtained from the knockout library. Geneticin-resistant transformants were screened for their ability to secrete acid phosphatase using the colorimetric pNPP reporter assay described above. Transformants that were positive for secreted acid phosphatase activity were further tested by Southern blotting and qPCR for the expression of *PHO4* and the Pho4-dependent genes, *PHO89*, *PHO84*, and *APH1* (Fig. S2 and S3). All primers used are listed in Table S1.

### Fluorescence microscopy.

Fungal cell fluorescence was viewed using a DeltaVision deconvolution microscope. To visualize Pho4 in the Pho4-mCherry-expressing strain constructed as described above, the cells were grown overnight in YPD, washed twice with water, resuspended in either phosphate-replete medium (MM-KH_2_PO_4_) or phosphate-deficient medium (MM-KCl) and incubated for 2 h at 30°C. To visualize fungal nuclei, 1 µl of Hoechst stain was added to 200 µl of cells for 5 min after 1-h incubation in either MM-KH_2_PO_4_ or MM-KCl.

### ^32^P_i_ uptake.

Fungal cells were grown overnight in YPD broth, washed with water, and resuspended in RPMI medium at an optical density at 600 nm (OD_600_) of 1 (Fig. 8C) or in YPD at an OD_600_ of 3 (Fig. S4A). Radioactive orthophosphate (^32^P_i_) (catalog no. NEX011002MC; PerkinElmer) was then added to the samples (1 to 2 µl/ml culture). For the experiments conducted in MM (P_i_-deficient medium [Fig. 1C]), YPD-grown cells were washed and incubated in MM for 2 h to trigger phosphate starvation. The OD_600_ of the cultures was then adjusted to 1, and ^32^P_i_ was added. For the MM-based experiments, ^32^P was premixed with cold phosphate (KH_2_PO_4_ at a final concentration of 0.1 mM) to prevent the immediate uptake of radioactive phosphate. The radiolabeled cultures were incubated as indicated in the figure legends and centrifuged. The amount of ^32^P in the cell pellets was then measured using a scintillation counter.

### Quantification of polyPs using a malachite green colorimetric assay.

Fungal cells were grown in RPMI-FBS medium buffered at pH 5.4 or 7.4 for 24 h at 30°C with shaking from a starting OD_600 _of 0.05. Isolation of RNA or polyphosphate chains (polyPs) and polyP quantification was performed as described in reference 12 with minor modifications. Briefly, 540 ng RNA was diluted in 1 M perchloric acid (total volume of 54 µl), and hydrolysis of the phosphodiesteric bonds was performed by incubating the samples for 30 min at 90°C. Thirty microliters of each sample was used for phosphate quantification using a malachite green phosphate assay kit (catalog no. MAK307; Sigma). The concentration of hydrolyzed orthophosphates was compared with a phosphate standard calibration curve.

### Ethics statement.

All animal procedures were covered by protocol 4254, which was approved by the Western Sydney Local Health District Animal Ethics Committee, and carried out in accordance with the guidelines from The National Health and Medical Research Council of Australia. All mice were maintained with environmental enrichment in specific-pathogen-free (SPF) conditions. All surgical procedures were performed on animals under transient anesthesia with 3% isoflurane, and all surgical procedures were conducted by trained personnel.

### Murine models of cryptococcosis.

Female C57BL/6 mice (20 to 22 g) were obtained from the Animal Resource Centre, Floreat Park, Western Australia, Australia. *Cryptococcus* strains were grown overnight in YPD broth, washed twice with phosphate-buffered saline (PBS), and resuspended in PBS. Prior to both intranasal and intravenous infection, mice were transiently anesthetized by inhalation of 3% isoflurane. For intravenous infection, fungal suspensions were injected via the retro-orbital plexus (5,000 cells in 200 μl PBS; seven mice in each infection group). For intranasal infection, 5 × 10^4^ (survival study with organ burden at the time of death) or 1 × 10^5^ (assessment of immune cell infiltrate) cells in 20 µl of PBS were delivered into the nares using a pipette as described previously (41). For the survival assays, 10 mice in each group were monitored daily and euthanized by CO_2_ asphyxiation when they had lost in excess of 20% of their preinfection weight or if showing debilitating symptoms of infection, i.e., loss of appetite, moribund appearance, or labored breathing. Differences in survival were determined using a Kaplan-Meier log rank test. For blood sampling, mice were anesthetized by inhalation of 3% isoflurane, and blood was collected from the retro-orbital plexus. For organ burden analysis, mice were sacrificed by CO_2_ inhalation on designated days postinfection or when they had lost 20% of the preinfection weight. Lungs and brain were then removed, weighed, and mechanically disrupted in 2 ml sterile PBS using a BeadBug (Benchmark Scientific). Serial dilutions of the organ samples were plated onto SDA agar plates and incubated at 30°C for 2 days. Colony counts were performed and adjusted to reflect the total number of CFU per gram of tissue.

### Assessment of immune cell infiltrate.

Mice were infected intranasally with the WT or *pho4*Δ strain (*n* = 5 per strain) as described above and euthanized 10 days postinfection. Euthanized animals were perfused with 10 ml PBS, and lungs were removed and placed in 1 ml of PBS with 2% fetal calf serum (FCS) (PBS–2%FCS). Single-cell suspensions of lung cells were prepared, and antibody staining for flow cytometric analysis was performed as published previously (42). Briefly, lungs were dissociated by mechanical disruption and incubated in RPMI 1640 supplemented with 2 mg/ml of DNase I (Sigma-Aldrich) and collagenase IV (Sigma-Aldrich) for 30 min at 37°C. Following single-cell dissociation through a 70-µm cell strainer (Miltenyi Biotec, Inc.), red blood cells were lysed with ACK lysis buffer (Life Technologies, Inc.), and total lung leukocytes were enumerated by trypan blue exclusion.

For flow cytometric analysis, 1 × 10^6^ lung cells were incubated for 30 min with UV LIVE/DEAD dye (Life Technologies, Inc.) and with the following antibodies in fluorescence-activated cell sorting (FACS) wash (PBS–2%FCS): CD4 (clone GK1.5), CD8 (53-6.7), B220 (RA3-6B2), I-A/I-E (M5-114.15.2), Ly6G (1A8), Ly6C (HK1.4), CD11b (M1/70), NK1.1 (PK136), Siglec-F (E50-2440), CD11c (N418), CD16/32 (clone 93), CD64 (X54-5/7.1), CD206 (C068C2), and F4/80 (BM8). Flow cytometry data acquisition was performed on an LSRII instrument using FACSDiva software (BD Biosciences), and all analysis was performed using FlowJo V10 (TreeStar). Cell populations were gated as described previously (42).

### PBMC-cryptococcus coculture.

Fungal cells were grown overnight in YPD broth, washed with water, and labeled with 0.5 mg/ml fluorescein isothiocyanate (FITC) (Sigma) in HEPES-buffered saline for 30 min. Excess stain was removed by washing the cells three times with HEPES-buffered saline, and the cells were resuspended in RPMI-FBS medium. Fungal cells (5 × 10^4^) were opsonized for 30 min in 25 µl of 75% human serum supplemented with 0.1 μl opsonizing antibody 18B7 directed against glucuronoxylomannan (GXM) (a gift from Arturo Casadevall, Johns Hopkins, MD). Fungal cell number was confirmed by plating appropriate dilution onto the SDA agar.

PBMCs were isolated from ~25 ml human blood using Ficoll-Paque Plus (GE Healthcare) following the manufacturer’s protocol, and resuspended in RPMI-FBS. PBMCs (5 × 10^5^) were mixed with 5 × 10^4^ cryptococci in 200 µl of RPMI-FBS and coincubated for 90 min (time zero) and overnight (24 h) at 37°C in 5% CO_2_. The interaction of PBMCs and cryptococci was visualized by DeltaVision fluorescence microscopy. To quantify the growth of cryptococcal cells in the presence and absence of PMBCs, the number of viable cells was assessed by plating serially diluted samples onto SDA agar. PBMCs were lysed in 0.05% SDS prior to plating.

### Histology.

Organs collected from infected mice were fixed in 4% paraformaldehyde (PFA) and embedded in paraffin. Transversal sections (4 µm) were prepared and stained with hematoxylin and eosin (H&E) or periodic acid-Schiff (PAS) for the detection of fungal cells. Sections were viewed under an Olympus ix71 microscope or a laser scanning confocal microscope (LSM 510 Meta laser scanning confocal system; Zeiss, Germany).

### Statistics.

Statistical analysis was performed using JMP statistical software (SAS Institute Inc.). For mouse studies, differences in survival were determined using a Kaplan-Meier log rank test. Virulence studies were further verified using a Fit model test to include the experimental time period. Analysis of CFU variance was determined using one-way analysis of variance (ANOVA) or Student’s *t* test. All other statistics were performed using a Student’s *t* test. All data are plotted to represent the mean ± standard deviation. Results were considered significant at a *P* of <0.05.
