# Supplementary material for: Pho4 Is Essential for Dissemination of Cryptococcus neoformans to the Host Brain by Promoting Phosphate Uptake and Growth at Alkaline pH
Source: mSphere. 2017 Jan 25;2(1):e00381-16. doi: 10.1128/mSphere.00381-16 (PMC5266496; doi:10.1128/mSphere.00381-16)

A

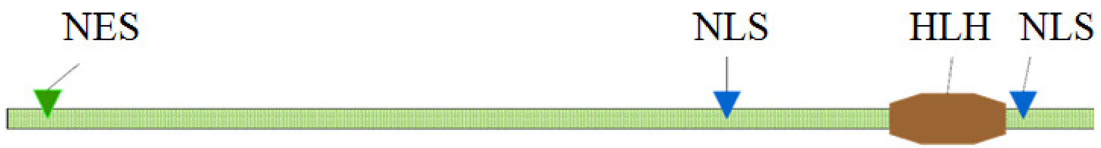

B

```
Nc_AAA33603       -SKRTSHKIAEQGRRNRINSALQEIATLLPKAPAKEGGDGDGDGHSSSGGGGGSGGADREDKR---EK--DKDKAGGGIPNSKASTVEMAIEYIKQLQKEVA
Fo_EMT61942       -SKRTSHKIAEQGRRNRINSALQVMAGLLP------GGD--------K-----TNLADEGDK-----K--DGKQAN--AQNSKASVVENAIVHMKSLEKEN-
Af_XP_747924      SSKRTNHKLAEQGRRNRINNALKEIESLIPSAFIQMKQTK-------------ENVASHVKGDKEKEK--EKAGAPT---ISKASTVELAIDYIKALKQELE
Ca_KHC37397       --KKASHKLAEQGRRNRMNNAVQELGRLIPQSY-----------------------HDEVS-------------IP-----SKATTVELASKYITALLKEVE
Sc_Pho4           --KRESHKHAEQARRNRLAVALHELASLIPAEWK----------------------QQNVS------------AAP-----SKATTVEAACRYIRHLQ----
Ptr_EDU50072      -SKRTSHKIAEQGRRNRINMALQEMQALLPSPQFG-------------------ATPDAKSP----ES--NAQNSN----NSKAAKVESAIEYIKQLKQEVS
CnA_CNAG_06751    --RKISHKAAEQKRRDSLKAGFDELRLLLPPINTEALDP-------LSG----EPIPGSSAPRLLPKSSLVPDDNPN-RGVSKVALLRFGNEYIGKLQ----
CnD_CNB00520      --RKISHKAAEQKRRDSLKAGFDELRLLLPPINTEALDP-------LSG----EPIPGSSAPRLLPKSSLVPDDNPN-RGVSKVALLRFGNEYIGKLQ----
                    :: .** *** **: :  ... :  *:*                                                   **.: :. .  ::  *
```

C

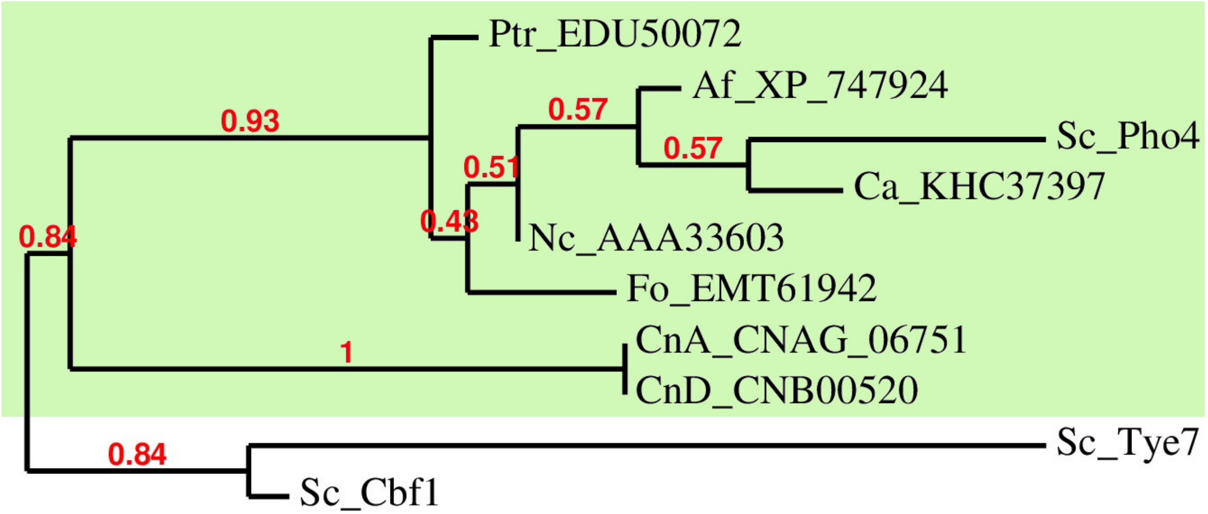

Supplement: FIG S1 [file sph001172224sf3.pdf]
