# Supplementary figures and images for: Pho4 Is Essential for Dissemination of Cryptococcus neoformans to the Host Brain by Promoting Phosphate Uptake and Growth at Alkaline pH
Source: mSphere. 2017 Jan 25;2(1):e00381-16. doi: 10.1128/mSphere.00381-16 (PMC5266496; doi:10.1128/mSphere.00381-16)

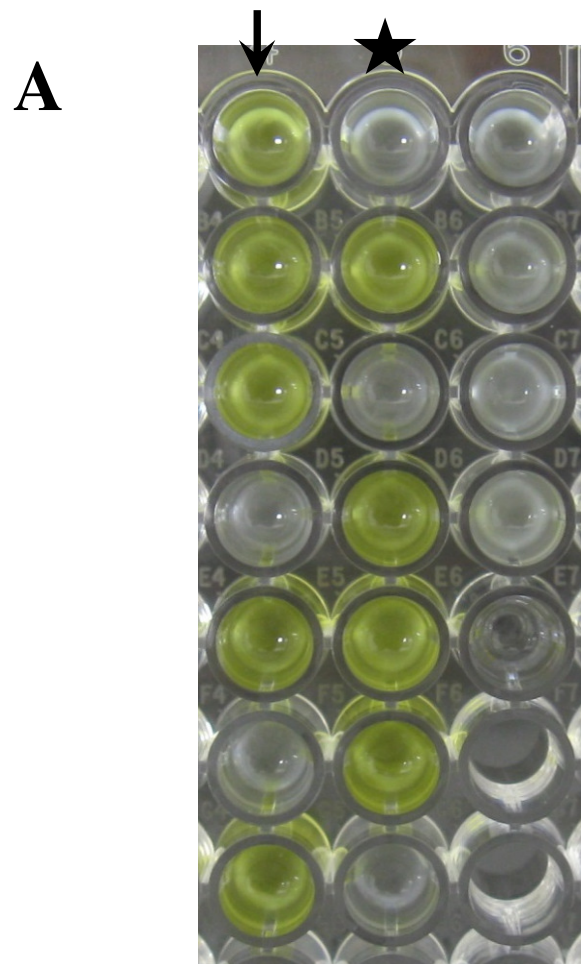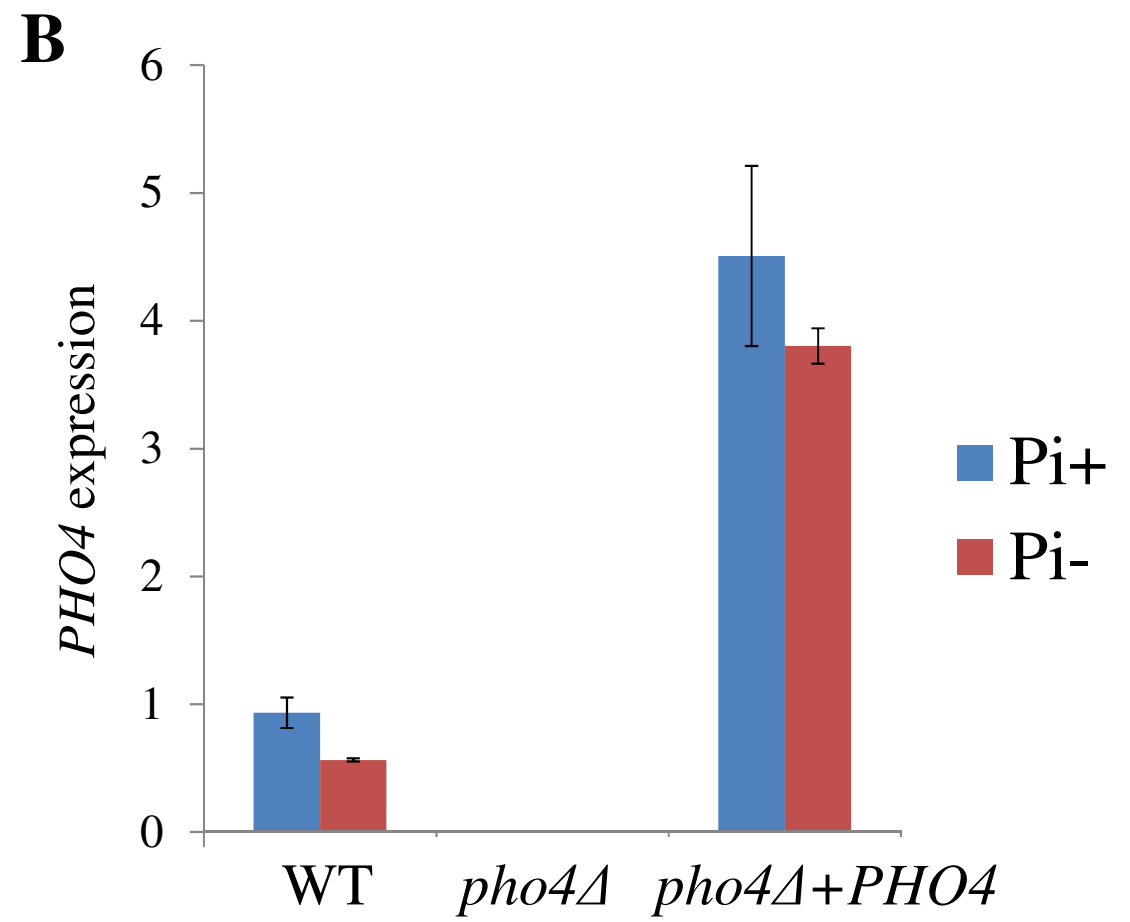

Supplement: FIG S2 [file sph001172224sf4.pdf]

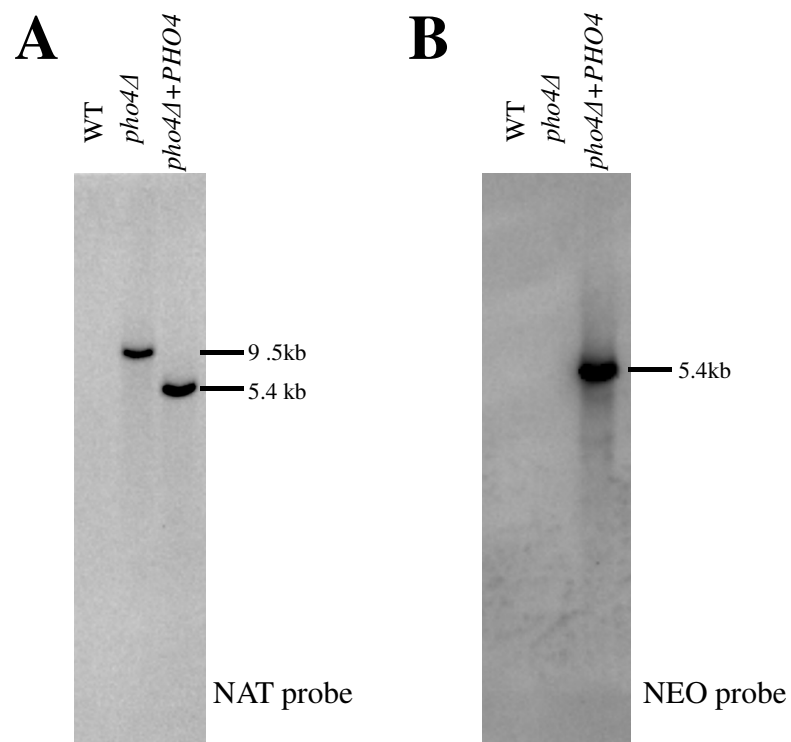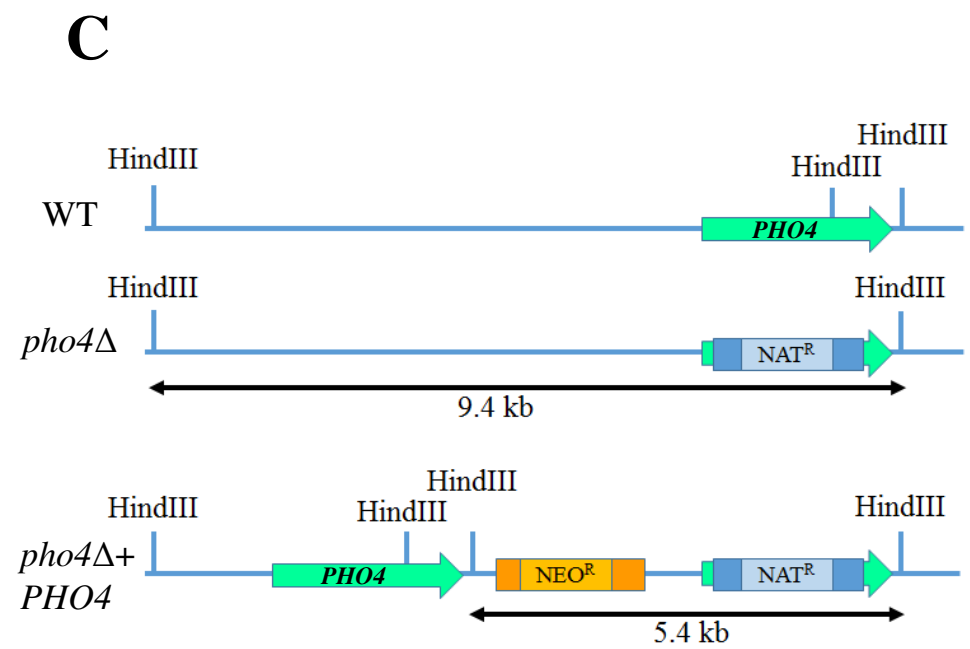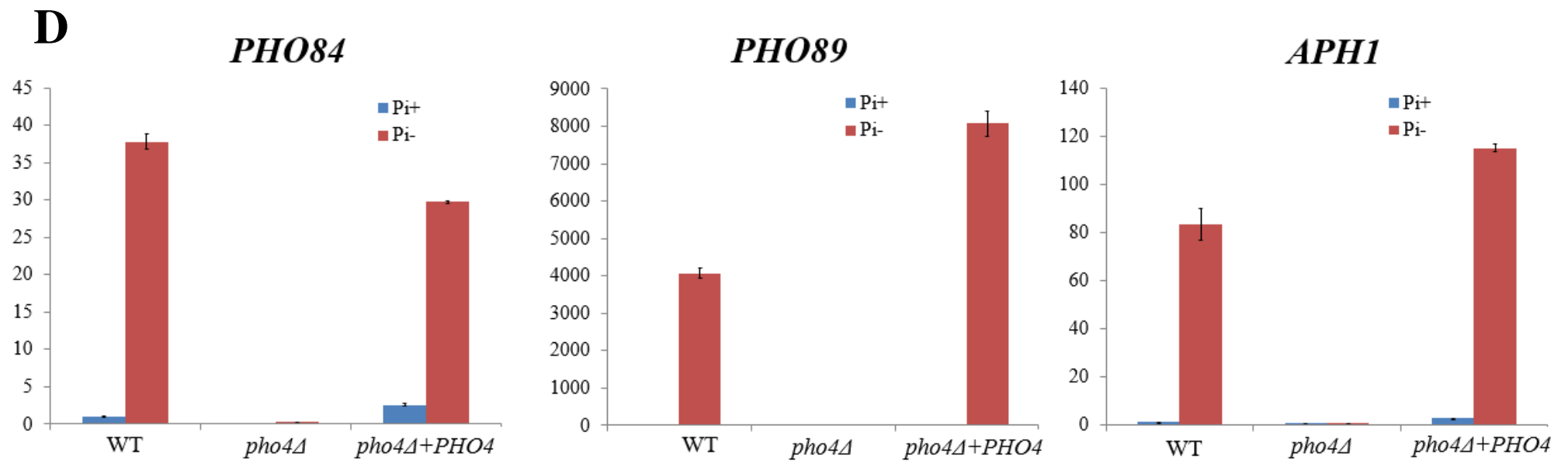

Supplement: FIG S3 [file sph001172224sf5.pdf]

**A**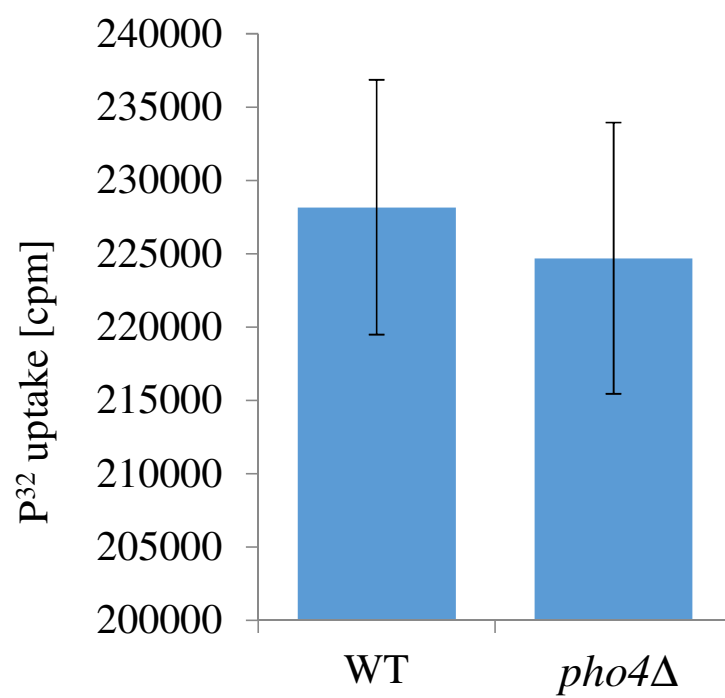**B**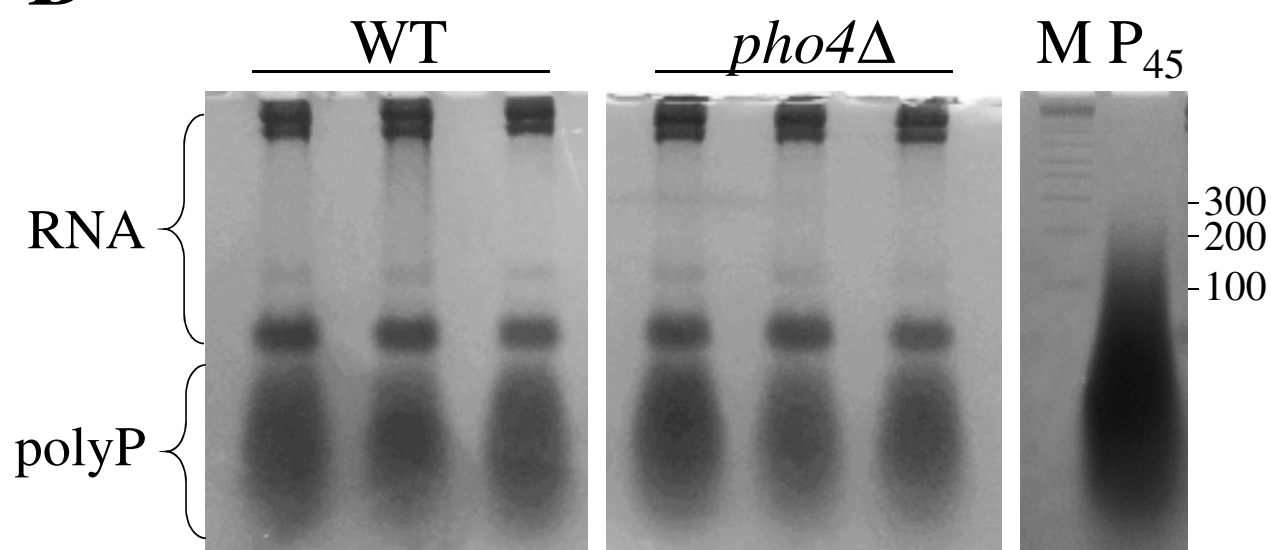

Supplement: FIG S4 [file sph001172224sf6.pdf]

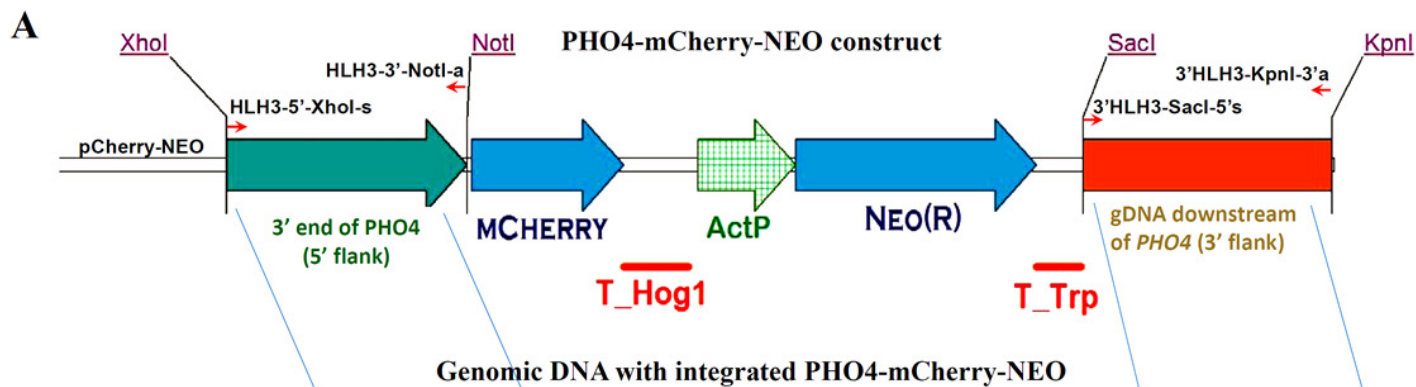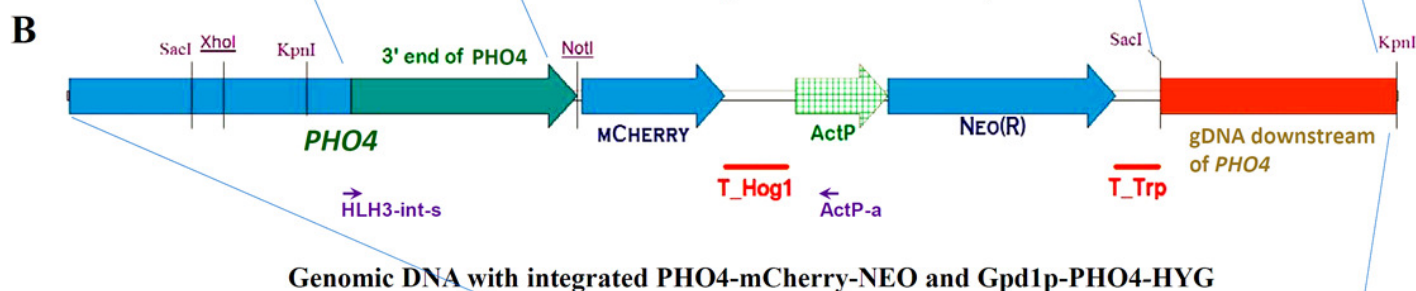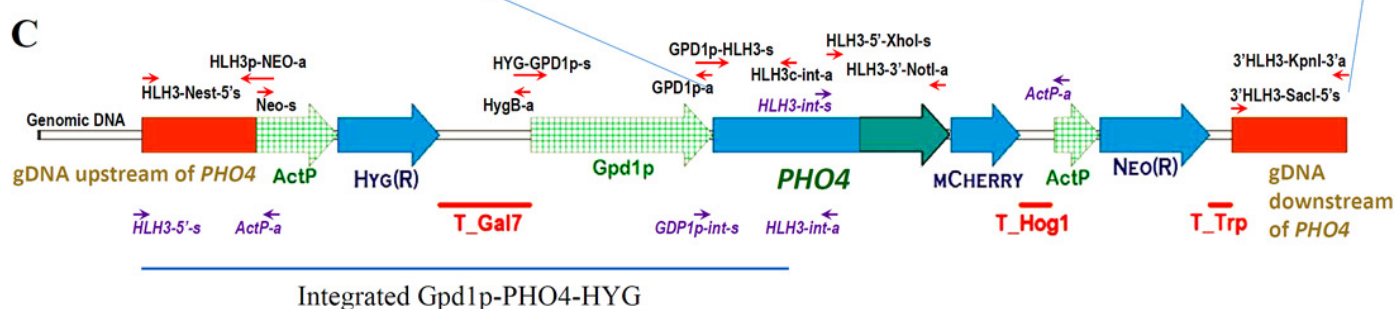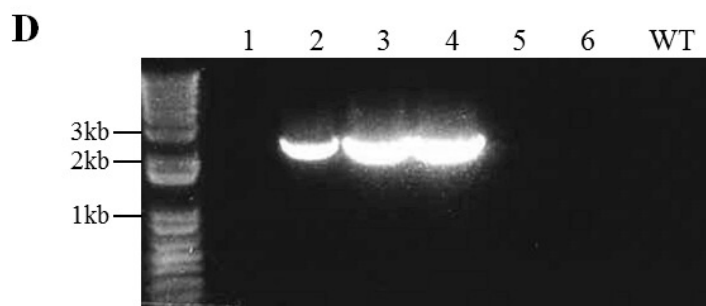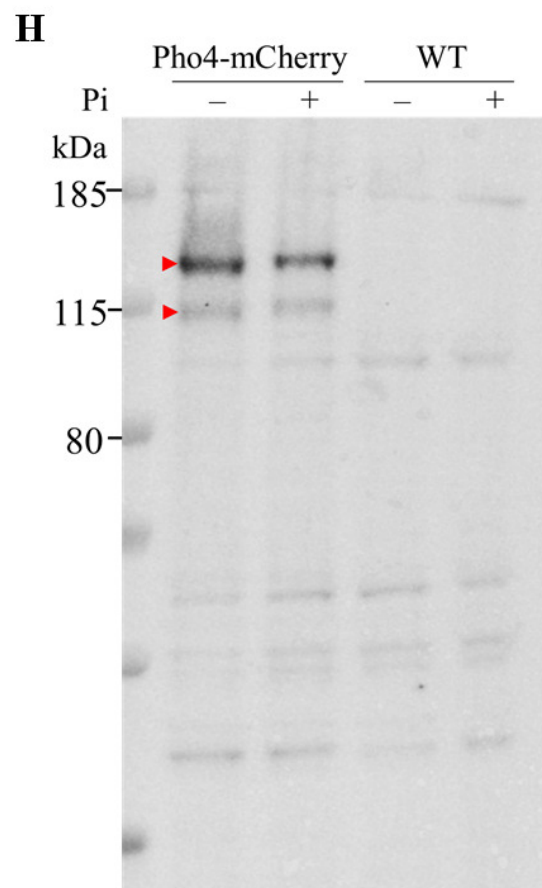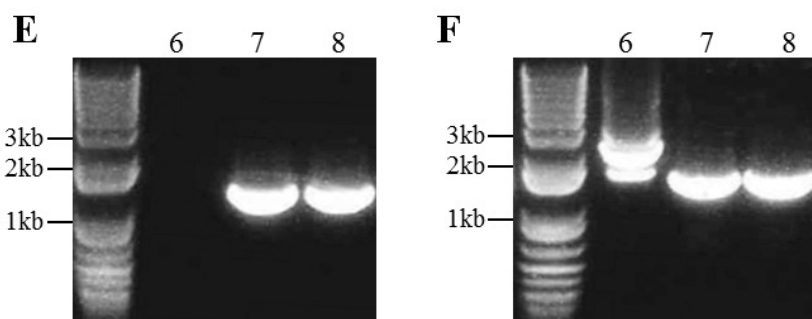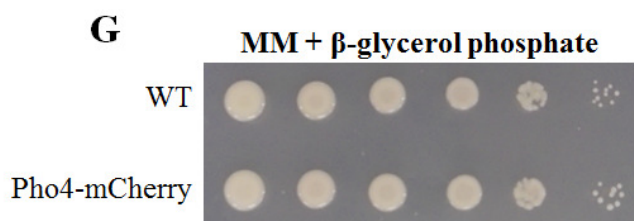

Supplement: FIG S5 [file sph001172224sf7.pdf]

**A**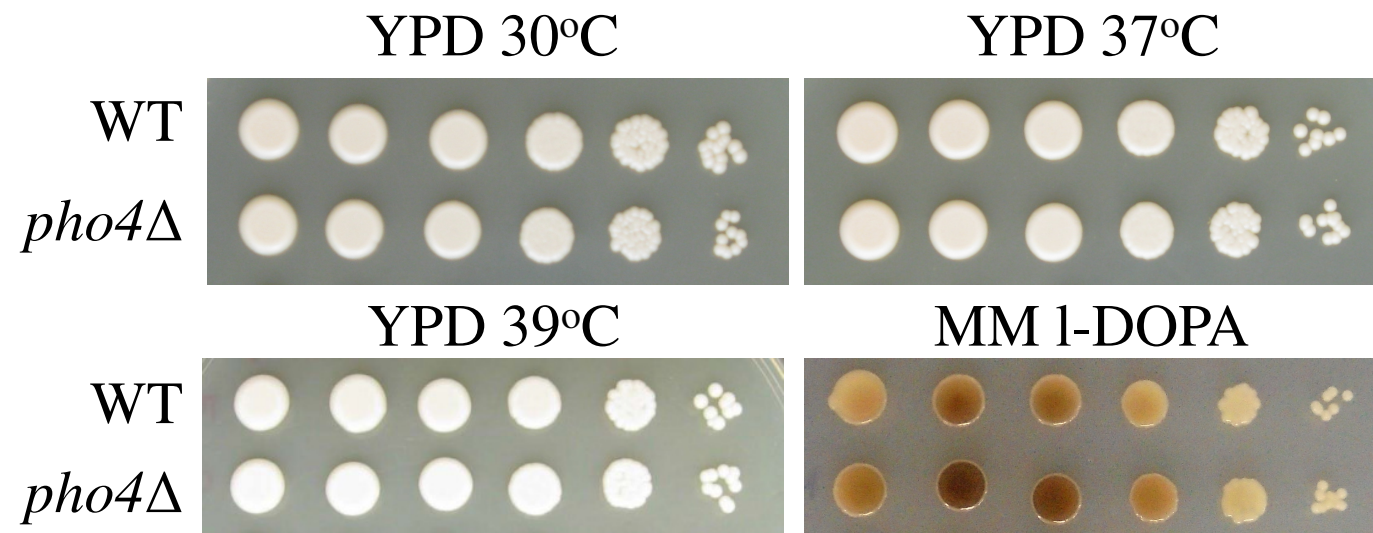**B**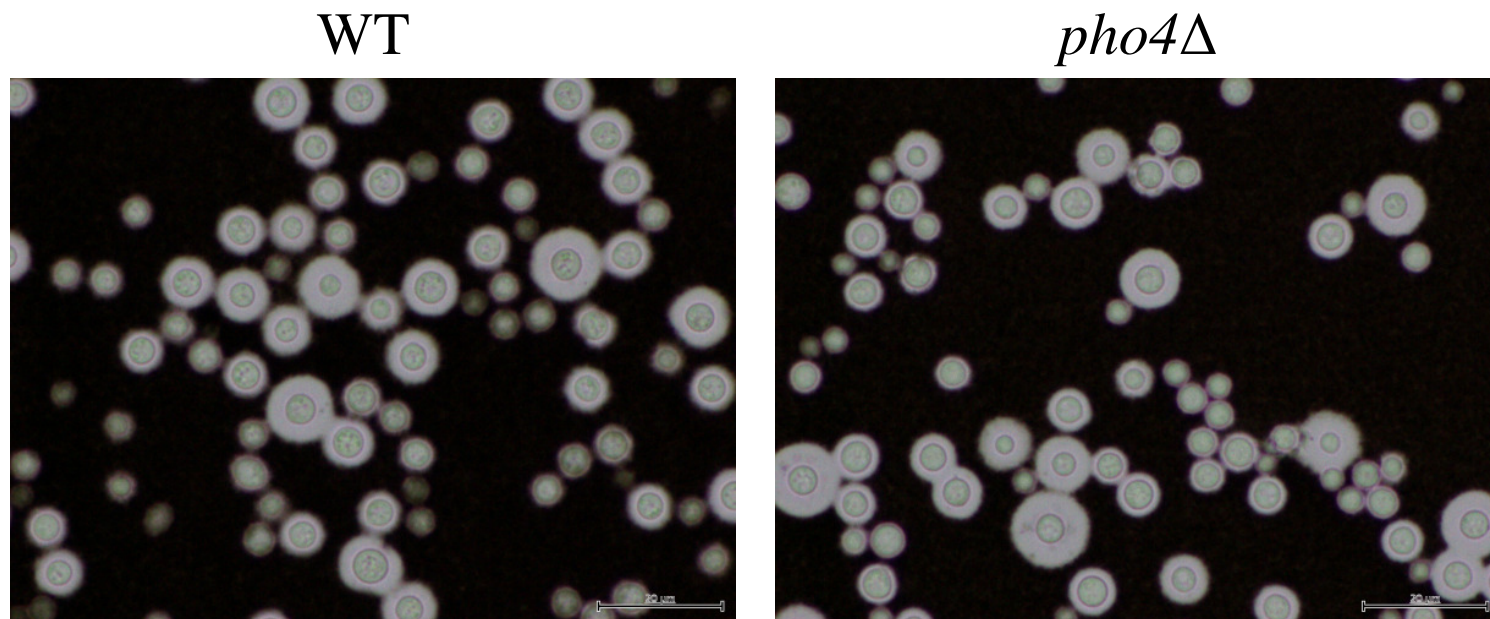

Supplement: FIG S6 [file sph001172224sf8.pdf]

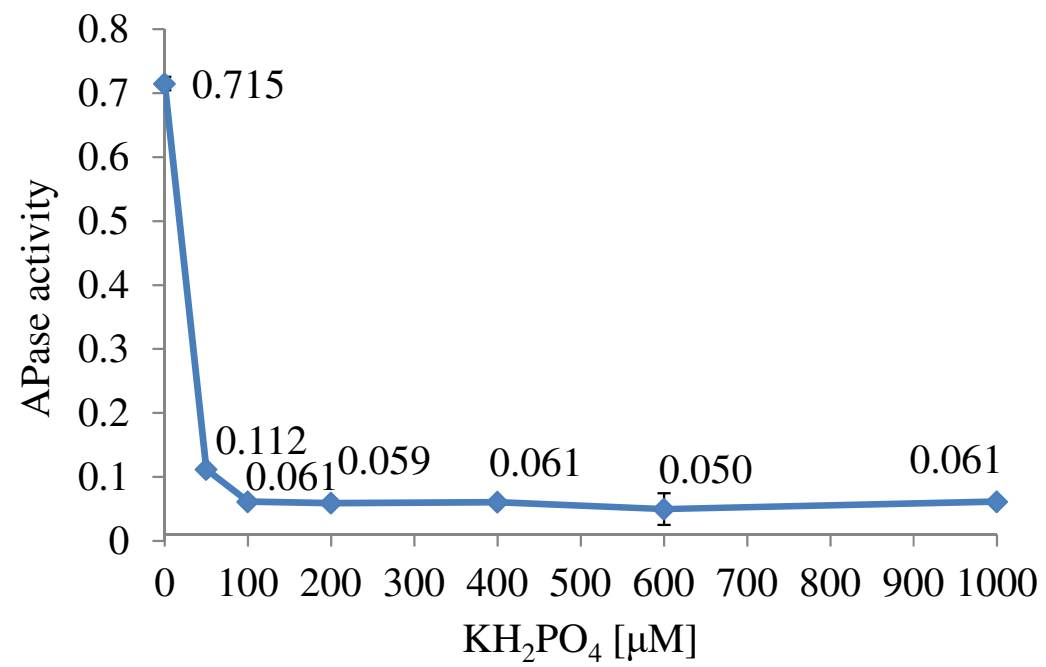

Supplement: FIG S7 [file sph001172224sf9.pdf]

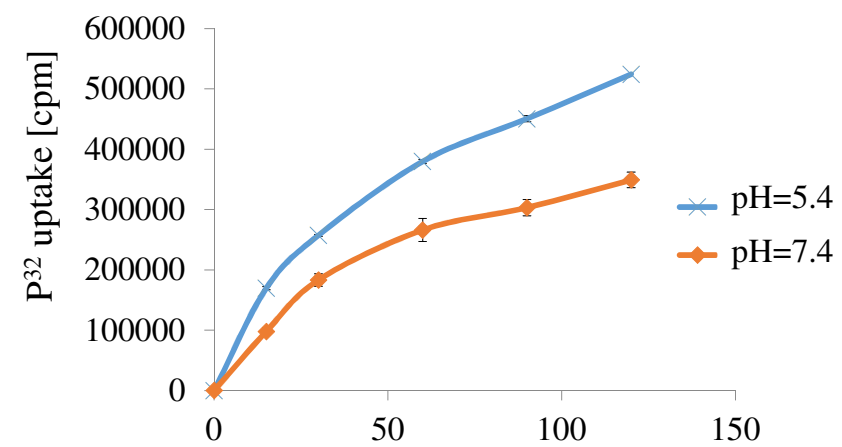

Supplement: FIG S8 [file sph001172224sf10.pdf]
