# Supplementary material for: Pho4 Is Essential for Dissemination of Cryptococcus neoformans to the Host Brain by Promoting Phosphate Uptake and Growth at Alkaline pH
Source: mSphere. 2017 Jan 25;2(1):e00381-16. doi: 10.1128/mSphere.00381-16 (PMC5266496; doi:10.1128/mSphere.00381-16)
Supplement: TABLE S1 [file sph001172224st2.docx]

**Table S1. Primers used in this study.**

| **Description** | **Name** | **Sequence** | **Name** | **Sequence** |
| --- | --- | --- | --- | --- |
| **qPCR** | | | | |
| PHO84 expression | CnPho84_frt | CCTACTCGTTACCGATCAACTG | CnPho84_rrt | AGTCTCGGGAAGAAGCAATG |
| PHO89 expression | CnPho89_frt | GTGCTCGGTAACAGACTGAC | CnPho89_rrt | ACGCTCGCCAGTTAATCG |
| PHO840 expression | CnPho840_frt | CCTTCCCGCCGTTATCTAC | CnPho840_rrt | GATACTCGTGTGCCCTACC |
| VTC4 expression | CnVtc4_frt | GATGCCGTCGGTATGGTTTC | CnVtc4_rrt | TAACAACGCCGCGCAAAG |
| ACT1 expression | ACT1-RTF | ATGGTATTGCCGACCGTATG | ACT1-RTR | CTCTTCGCGATCCACATCTG |
| PHO4 expression | HLH3-int-s | TTCAAATCCCGTCCTTCGCCG | HLH3-int-a | CGTTCATAGCGGCAGGAGGCAT |
| APH1 expression | APH qRT-s v2 | CCTACTTCCCACTCAACCAATCCA | APH qRT-a v2 | CCTGCGAAGCCACAAACGAA |
| APH2 expression | 06967qRT L1 | TCTCGGTCACTCTGCCTTCT | 06967qRT R1 | CGCCTTAGCAGGAGCATATC |
| APH3 expression | 02681qRT L1 | GACCGATTCTGCTCCTCAAG | 02681qRT R1 | GGGACGATTCGGGAAAGA |
| APH4 expression | 06115qRT L1 | AAAGCAATGCCACGGTAAAC | 06115qRT R1 | CCCAAGCCTGTAGATTGCAT |
| PHO81 expression | GDE1 s | AAGAAGGTAGGAAGGGAGAGCGG | GDE1 a | ATGAAGATTGACGGGAGACGCC |
| GDE2 expression | GDE2 s | GGGGTATTGCCAGTGTCATTTCAGA | GDE2 a | TATTCCGTTTCTTCCTTGGCGAG |
| BTA1 expression | BTA1 s | CCCATTCCAACGCTTTCTACTCTCA | BTA1 a | AGCGACTCATCAGGAAGACCCC |
| **GDP1p-PHO4-mCherry construct** | | | | |
| PHO4-mCherry-Neo(R) construct. *PHO4* genomic sequence, 3' end | HLH3-5'-XhoI-s | gccCTCGAGACCGCAACTCTGGCTCTGGCA | HLH3-3'-NotI-a | ccgGCGGCCGCTCCTTCATCTCCGTTCC |
| PHO4-mCherry-Neo(R) construct. Downstream region of *PHO4* | 3'HLH3-SacI-5's | ggcGAGCTCTCAACAAAGAATAATCAGGAGCCAA | 3'HLH3-KpnI-3'a | cgcGGTACCTCCTACTGTCGGTGGTGGTCTCAA |
| Verification of PHO4-mCherry-Neo(R) transformants | HLH3-int-s | TTCAAATCCCGTCCTTCGCCG | ActP-a | TGTTGTTACCATCATCCTCTCCTC |
| Amplification of upstream region of *PHO4* genomic sequence | HLH3-Nest-5's | CGACGACACAAGAGGAACGAAGGT | HLH3p-NEO-a | CTCCAGCTCACATCCTCGCAGCCGATGCCACTCCACGAACGT |
| Hyg(R) cassette amplification | Neo-s | CTGCGAGGATGTGAGCTGGAG | HygB a | TCTCTATACGGCGATTGGCGGA |
| GPD1p amplification | HYG-GPD1p-s | TCCGCCAATCGCCGTATAGAGAGCGCGAACGCCATCCTCAAA | GPD1p-a | TGTATTTATGCAAGTATACTCCTAGAAGG |
| PHO4 genomic sequence, 5' end | GPD1p-HLH3-s | AGTATACTTGCATAAATACAATGATCAGCACAATGTCAACCT | HLH3c-int-a | ATTGTTGGCTGTGACGGCTGAC |
| Overlap PCR to fuse all 4 fragments to create GPD1p-PHO4-HYG construct | HLH3-Nest-5's | CGACGACACAAGAGGAACGAAGGT | HLH3c-int-a | ATTGTTGGCTGTGACGGCTGAC |
| Verification of Hyg(R)-GPD1p-PHO4 transformants, external 3' recombination | GPD1p-int-s | TGGAACGAGGCGGGAACAAAC | HLH3-int-a | CGTTCATAGCGGCAGGAGGCAT |
| Verification of Hyg(R)-GPD1p-PHO4 transformants, external 5' recombination | HLH3-5'-s | CCTTGCCTGAATCCTTGCCTGAA | ActP-a | TGTTGTTACCATCATCCTCTCCTC |
| **Construction of the *PHO4* reconstitution vector** | | | | |
| Amplification of *PHO4* complete ORF with upstream and downstream region | HLH3-5'-s | CCTTGCCTGAATCCTTGCCTGAA | HLH3t-a | GCGGCAAGTATGAATGGGAAAG |
| Amplification of Neo(R) cassette | (HLH3t)-NEO-s | CTTTCCCATTCATACTTGCCGC CTGCGAGGATGTGAGCTGGAG | Neo-a | GGAGCCATGAAGATCCTGAGG |
| Overlap PCR to create PHO4-NEO reconstitution construct | HLH3-5'-s | CCTTGCCTGAATCCTTGCCTGAA | Neo-a | GGAGCCATGAAGATCCTGAGG |
| Verification by qPCR to test *PHO4* expression | HLH3-int-s | TTCAAATCCCGTCCTTCGCCG | HLH3-int-a | CGTTCATAGCGGCAGGAGGCAT |
