# Supplementary material for: Pho4 Is Essential for Dissemination of Cryptococcus neoformans to the Host Brain by Promoting Phosphate Uptake and Growth at Alkaline pH
Source: mSphere. 2017 Jan 25;2(1):e00381-16. doi: 10.1128/mSphere.00381-16 (PMC5266496; doi:10.1128/mSphere.00381-16)
Supplement: TEXT S1 [file sph001172224s1.docx]

**Supplementary methods**

**Quantification of polyPs by metaphore gel electrophoresis**

*C. neoformans* was grown overnight in YPD, pelleted by centrifugation and resuspended in fresh YPD (OD_600_=3). The cultures were incubated for 8 hours at 30^o^C with shaking. RNA and polyphosphates were isolated as described for *S. cerevisiae* in (1), except that cryptococcal cells were broken using a bead beater (4 cycles of beating for 30 sec with 1 minute rest intervals). To quantify polyPs, 14 µg of RNA in TBA buffer and 30% glycerol was separated by electrophoresis on a 3% metaphore gel in TBA buffer. 20 µg of sodium phosphate glass P45 (Sigma, S4379) and 100 bp DNA ladder (Ferments) were used as markers. To visualize DNA/RNA/polyPs, the gel was stained with 0.01% Toluidine Blue for 30 minute, followed by distaining in water.

**Induction of extracellular acid phosphatase (APase) activity at different concentrations of free phosphate**

WT H99 cells were grown overnight in YPD broth, washed twice with water and resuspended at OD_600_=1 in MM supplemented with KH_2_PO_4_ at different concentrations. The cells were incubated for 3 hours at 30^o^C with shaking and the APase activity quantified as described in “Screening for transcription factors involved in phosphate homeostasis in *C. neoformans*”. APase activity values were normalized to fungal growth as determined by measuring OD_600_.

1. **Anti-mCherry Western Blot**
2. *C. neoformans* cells were grown overnight in YPD, washed twice with water and resuspended at an OD_600_ =1 in either phosphate-replete (MM-KH_2_PO_4_) or phosphate-deficient (MM-KCl) media. Cells were incubated at 30°C for 3 hours, pelleted by centrifugation and snap-frozen in liquid nitrogen. Cells were thawed in Trizol (Ambion), mixed with glass beads (425 µm – 600 µm) and disrupted with bead-beating: 4 x 30 seconds with a MiniBeadbeater-8 cell disrupter (Daintree Scientific, TAS, Australia). Protein extraction with Trizol was then carried out according to the manufacturer’s instructions. The final protein pellet was dissolved in NuPAGE® LDS Sample Buffer (4X) and NuPAGE® Sample Reducing Agent (10X). Protein was loaded onto a NuPAGE® Novex® 4-12% Bis-Tris Protein Gel, and separated in NuPAGE® MOPS SDS Running Buffer. Proteins in the gel were then transferred onto PVDF membrane using a Towbin buffer system (25 mM Tris-HCl pH 8.3, 192 mM glycine, 20% (v:v) methanol). PVDF membrane was blocked with 5% Blotto non-fat milk in TBST (50 mM Tris-Cl, pH 7.5, 150 mM NaCl, 0.5% Tween-20) and then incubated with a 1:100 dilution of anti-mCherry antibody (Abcam, Cat. No. ab183628) in TBST at 4°C overnight. The membrane was washed 3 times with TBST followed by incubation with a 1:5000 dilution of anti-rabbit IgG-HRP (Amersham ECL Rabbit IgG, HRP-linked whole Ab, Cat. No. NA934-100UL) for 1 hour. The membrane was again washed with TBST and visualized using enhanced chemiluminescence on a ChemiDoc™ MP System (Bio-Rad Laboratories, Inc.)

**Southern Blot**

1. WT, *pho4*Δ and *pho4*Δ+*PHO4* strains were grown overnight in YPD and pelleted by centrifugation. Cells were resuspended in 300 µl of Triton SDS lysis buffer (2% v/v Triton X-100, 1% w/v SDS, 100 mM NaCl and 1 mM Na_2_EDTA), and 300 µl of phenol:chloroform:isoamyl-alcohol (25:24:1) and 300 µl of glass beads (425 µm – 600 µm) were added. Vortexing was carried out for 2 minutes, followed by centrifugation at maximal speed for 5 minutes to allow phase separation. The upper phase was transferred to a fresh tube and mixed with an equal volume of isopropanol to precipitate DNA. Following centrifugation for 30 min, the genomic DNA pellet was washed once with 75% ethanol and air-dried briefly before being dissolved in nuclease-free water. Genomic DNA (15 µg) was digested with HindIII. The digest was separated on a 0.8% Tris/Borate/EDTA gel and transferred to a nitrocellulose membrane by capillary transfer. The Nat^r^ and Neo^r^ probes were labelled with digoxigenin (DIG) using PCR and the (DIG)-UTP labelling kit (PCR DIG Probe Synthesis Kit; Roche, Cat. No. 636 090 910) according to the manufacturer’s instructions). The Nat^r^ probe was generated using the primer pair NAT_Sth_s (CCGCCACTCTTGACGACACG) and NAT_Sth_a (ATGCTCATGTAGAGCGCCTGCT) (2). The Neo^r^ probe was amplified from pJAF1 using the primer pair NEO_Sth_s (GATTGCACGCAGGTTCTCCG) and NEO_Sth_a (CGATAGAAGGCGATGCGCTG) (3). Following DNA transfer, the nitrocellulose membrane was incubated with DIG-labelled probe (2 µl/ml of DIG Easy Hyb™ buffer (Roche)) overnight at 55°C in a hybridization oven. Probe hybridization was detected with DIG High Prime DNA Labelling and Detection Starter Kit II (Roche, Cat. No. 11 585 614 910) using the DIG Wash and Block Buffer Set (Roche, Cat. No. 11 585 762 111).

**References**

1. Lonetti A, Szijgyarto Z, Bosch D, Loss O, Azevedo C, Saiardi A. Identification of an evolutionarily conserved family of inorganic polyphosphate endopolyphosphatases. The Journal of biological chemistry. 2011;286(37):31966-74.

2. Idnurm A, Reedy JL, Nussbaum JC, Heitman J. Cryptococcus neoformans virulence gene discovery through insertional mutagenesis. Eukaryotic cell. 2004;3(2):420-9.

3. Fraser JA, Subaran RL, Nichols CB, Heitman J. Recapitulation of the sexual cycle of the primary fungal pathogen Cryptococcus neoformans var. gattii: implications for an outbreak on Vancouver Island, Canada. Eukaryotic cell. 2003;2(5):1036-45.
